# Genome-wide association mapping reveals potential novel loci controlling stripe rust resistance in a Chinese wheat landrace diversity panel from the southern autumn-sown spring wheat zone

**DOI:** 10.1186/s12864-020-07331-1

**Published:** 2021-01-07

**Authors:** Yuqi Wang, Can Yu, Yukun Cheng, Fangjie Yao, Li Long, Yu Wu, Jing Li, Hao Li, Jirui Wang, Qiantao Jiang, Wei Li, Zhien Pu, Pengfei Qi, Jian Ma, Mei Deng, Yuming Wei, Xianming Chen, Guoyue Chen, Houyang Kang, Yunfeng Jiang, Youliang Zheng

**Affiliations:** 1 Triticeae Research Institute, Sichuan Agricultural University, Wenjiang, Chengdu, Sichuan 611130 P. R. China; 2 State Key Laboratory of Crop Gene Exploitation and Utilization in Southwest China, Wenjiang, Chengdu, Sichuan 611130 P. R. China; 3 College of Agronomy, Sichuan Agricultural University, Wenjiang, Chengdu, Sichuan 611130 P. R. China; 4 US Department of Agriculture, Agricultural Research Service, Wheat Health, Genetics and Quality Research Unit; and Department of Plant Pathology, Washington State University, Pullman, WA 99164-6430 USA

**Keywords:** Chinese wheat landrace, Southern China, Stripe rust resistance, GWAS

## Abstract

**Background:**

Stripe rust, caused by the fungal pathogen *Puccinia striiformis* f. sp. *tritici* (*Pst*), is a serious foliar disease of wheat. Identification of novel stripe rust resistance genes and cultivation of resistant cultivars are considered to be the most effective approaches to control this disease. In this study, we evaluated the infection type (IT), disease severity (DS) and area under the disease progress curve (AUDPC) of 143 Chinese wheat landrace accessions for stripe rust resistance. Assessments were undertaken in five environments at the adult-plant stage with *Pst* mixture races under field conditions. In addition, IT was assessed at the seedling stage with two prevalent *Pst* races (CYR32 and CYR34) under a controlled greenhouse environment.

**Results:**

Seventeen accessions showed stable high-level resistance to stripe rust across all environments in the field tests. Four accessions showed resistance to the *Pst* races CYR32 and CYR34 at the seedling stage. Combining phenotypic data from the field and greenhouse trials with 6404 markers that covered the entire genome, we detected 17 quantitative trait loci (QTL) on 11 chromosomes for IT associated with seedling resistance and 15 QTL on seven chromosomes for IT, final disease severity (FDS) or AUDPC associated with adult-plant resistance. Four stable QTL detected on four chromosomes, which explained 9.99–23.30% of the phenotypic variation, were simultaneously associated with seedling and adult-plant resistance. Integrating a linkage map of stripe rust resistance in wheat, 27 QTL overlapped with previously reported genes or QTL, whereas four and one QTL conferring seedling and adult-plant resistance, respectively, were mapped distantly from previously reported stripe rust resistance genes or QTL and thus may be novel resistance loci.

**Conclusions:**

Our results provided an integrated overview of stripe rust resistance resources in a wheat landrace diversity panel from the southern autumn-sown spring wheat zone of China. The identified resistant accessions and resistance loci will be useful in the ongoing effort to develop new wheat cultivars with strong resistance to stripe rust.

**Supplementary Information:**

The online version contains supplementary material available at 10.1186/s12864-020-07331-1.

## Background

Wheat (*Triticum aestivum*) is an important cereal crop worldwide and is a central pillar of global food security [1, 2]. In the coming decades, wheat production must increase more rapidly to keep pace with continued population growth [3]. However, to increase yield stably under climate change and biotic stress is an extreme challenge [4, 5]. Stripe rust, caused by the pathogenic fungus *Puccinia striiformis* f. sp. *tritici* (*Pst*), is a serious foliar disease of wheat that poses an increasing threat to wheat production worldwide [1]. The disease develops in wheat-producing areas with hypothermal and moist environments during the growing season, especially in China, which has experienced the largest wheat stripe rust epidemics by area in the world [6, 7]. The nationwide severe epidemics of wheat stripe rust in 1950, 1964, 1990 and 2002 caused substantial reductions in wheat yield [8]. In 2017, the stripe rust epidemic affected 1.65 million ha in 12 provinces [9]. Stripe rust is a critical constraint to wheat production and losses in grain yield can attain 40 to 100% under severe infections [10]. To reduce losses, appropriate application of fungicides is effective to control the disease. However, the effects of the high cost of fungicides and environmental concerns must be considered [11]. As a result of changes in the predominant races and emergence of new races, many wheat cultivars have become susceptible to stripe rust, thus accelerating the cultivar turnover frequency [7]. Mining of novel genetic resources and the breeding of disease-resistant cultivars is an effective, economic and environmentally friendly strategy to control stripe rust in wheat [7, 12].

Stripe rust resistance can be classified as all-stage resistance (ASR; also termed seedling resistance) or adult-plant resistance (APR) based on the growth stage of the plant [13]. The resistance genes can be classified as race-specific or race non-specific according to their effectiveness against different *Pst* races. Generally, race-specific resistance is expressed at all growth stages (from the seedling to the adult-plant stages) and thus belong to ASR. Wheat cultivars that carry these genes may become susceptible when new or rare pathogen races arise [14]. In contrast, genes conferring APR are usually race non-specific [15]. Combining APR and ASR genes is an important approach to develop new wheat cultivars with adequate durable resistance [11, 16, 17].

To date, 83 *Yr* genes for stripe rust resistance have been formally designated (*Yr1* to *Yr83*) and more than 100 temporarily named *Yr* genes or quantitative trait loci (QTL) have been reported [18–20]. However, many of these resistance genes are ineffective against newly prevalent *Pst* races or are not yet widely incorporated in wheat cultivars in China and elsewhere [21, 22]. As an example, *Yr9* was widely used in Chinese wheat breeding since the 1960s [8, 23]. A new *Pst* race CYR29 (Chinese yellow rust 29 with virulence to *Yr9*) was detected in 1985, resulting in yield losses of 2.65 million tonnes in 1990 [8]. Similar consequences were observed with the emergence and prevalence of the races CYR31, CYR32 and CYR33, resulting in loss of stripe rust resistance in many wheat cultivars (including Fan 6, Kangyin 655, Suwon 11 and their derivative cultivars) [8]. The race CYR34 emerged in 2009 and has become the main source of virulence against Guinong 22 and its derivative cultivars carrying the *Yr24/Yr26* locus [24]. At present, CYR32 and CYR34 are the most virulent and predominant races in China [9, 24]. Accordant with the aphorism “Rust never sleeps” [25], there is an ongoing need to search for novel sources of genetic resistance to stripe rust.

China is considered to be a unique epidemiological zone and the largest independent epidemic region [1]. Wheat stripe rust most frequently affects the winter wheat production areas in Northwest, Southwest and North China and the spring wheat growing areas in Northwest China [23]. There is considerable diversity in epidemiological conditions among the wheat-growing areas in China [26]. Overall, the region of southern Gansu and northwestern Sichuan was considered to be a “center of origin for virulence” [8]. Identification and utilization of novel sources of resistance genes are essential for improvement of stripe rust resistance in wheat breeding in this zone. Wheat landraces have been selected by farmers over many years to adapt to local environmental conditions [27]. Such landraces harbor great diversity of genes that respond to abiotic and biotic stresses and influence traits such as growth habit, cold, heat or drought tolerance, early growth vigor, competitiveness with weeds, and disease tolerance [27]. These genes may be important resources useful for stripe rust resistance breeding [12, 20, 28–31]. However, relatively few studies have investigated genetic diversity and stripe rust resistance in wheat landraces from the southern autumn-sown spring wheat zone of China.

Genome-wide association study (GWAS) is an effective approach to investigate complex phenotypic traits and to identify loci associated with target traits [32]. GWAS has been widely used to study agronomically important traits of a variety of crops, including maize, soybean, rice, cotton and wheat [33–37]. In addition, GWAS has been used to identify the genes underlying resistance to stripe rust in wheat [20, 38–40]. In the present study, 143 common wheat landrace accessions from the southern autumn-sown spring wheat zone of China were evaluated for resistance to *Pst* at the seedling and adult-plant stages in multiple years and field locations. We assessed the genetic diversity, population structure and linkage disequilibrium (LD) patterns of the accessions based on Diversity Arrays Technology sequencing (DArT-seq) and simple sequence repeat (SSR) markers and identified genomic regions controlling stripe rust resistance for utilization in wheat breeding.

## Results

### Analysis of stripe rust response

**Fig. 1:**
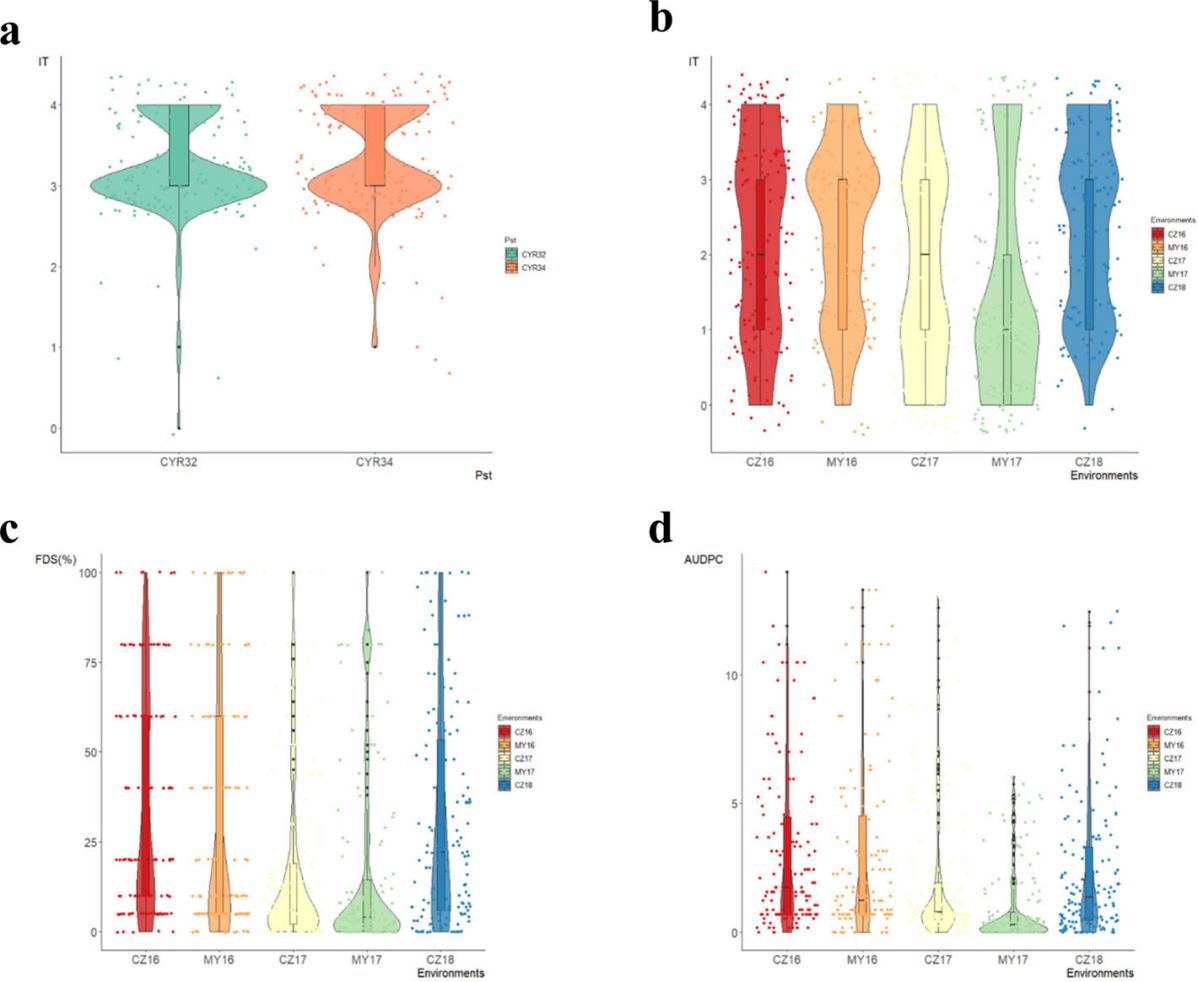
Box plot, violin plot and raw data points distributions of IT (**a**) evaluated in the seedling stage for CYR32 and CYR34; At the adult plant stage, IT (**b**), FDS (**c**) and the AUDPC (**d**) evaluated against *Pst* of mixed races in five environments. Tests at Chongzhou from the year 2016 to 2018 was referred to as CZ16, CZ17 and CZ18; at Mianyang from the year 2016 to 2017 referred to as MY16 and MY17, respectively

To characterize seedling resistance to stripe rust, we recorded the infection type (IT) response to the *Pst* races CYR32 and CYR34 at the seedling stage for the wheat landrace panel. The susceptible check Mingxian 169 was rated with IT = 4 for the two races tested. The majority of accessions in this panel showed a high frequency of susceptibility to CYR32 (95.8%) and CYR34 (93.7%), respectively. Based on the IT, four accessions (IT ≤2) including Lushanmai (AS661605), Yuqiumai (AS661657), Zhenixiaomai (AS661777) and Guangtoumai (AS661671) were resistant to both the *Pst* races (Fig. 1a, Additional file 1).

The responses of the 143 wheat landraces to mixed races of *Pst* were evaluated in five environments in the field (designated CZ16, CZ17, CZ18, MY16 and MY17). Based on BLUP values, a Pearson correlation analysis revealed significant correlations (*P* < 0.01) for IT, final disease severity (FDS) and area under the disease progress curve (AUDPC) that were observed among the five environments at the adult-plant stage, with correlation coefficients ranging from 0.58 to 0.89, 0.57 to 0.89 and 0.60 to 0.92, respectively (Additional file 2). The *H*^2^ values for IT, FDS and AUDPC were high across the five environments and BLUP values; the *H*^2^ values were 93.98, 94.07 and 94.02%, respectively (Table 1). The panel showed a higher frequency of resistance in the field environments than that observed in the seedling tests. With regard to IT (≤ 2), 48.3–75.5% of the accessions displayed resistance to the mixed *Pst* races in all five environments at the adult-plant stage (Fig. 1b, Additional file 1). Similarly, 63.6–89.5% of the accessions displayed resistance with low FDS values (< 60%) under the five environments (Fig. 1c, Additional file 1). Across the five environments, the phenotypic performance of the panel varied from 0 to 14 for AUDPC (Fig. 1d, Additional file 1). Seventeen accessions showed stable high-level resistance to stripe rust across all environments under field tests. These accessions originated from Sichuan (6), Yunnan (6), Gansu (3), Guizhou (1) and Shaanxi (1) (Additional file 1), respectively. Among these accessions, Lushanmai (from Sichuan) and Guangtoumai (from Guizhou) showed stable resistance to the *Pst* races CYR32 and CYR34 at the seedling stage and resistance in all field environments. In addition, Bendiyoumangxiaomai (from Yunnan) and Liulengmai (from Guizhou) likely showed ASR resistance to a single *Pst* race (CYR32 or CYR34) (Additional file 1).

**Table 1:** Summary of the stripe rust response among five environments

Traits	Trials	Minimum	Maximum	Mean	Heritability (%)
IT ^a^	CZ16	0	4	2.22	93.98
	MY16	0	4	2.28	
	CZ17	0	4	1.80	
	MY17	0	4	1.49	
	CZ18	0	4	2.40	
	BLUP	0.24	3.85	2.09	
FDS ^b^ (%)	CZ16	0	100	34.62	94.07
	MY16	0	100	29.86	
	CZ17	0	100	17.87	
	MY17	0	100	16.24	
	CZ18	0	100	31.72	
	BLUP	3.59	87.51	26.64	
AUDPC ^c^	CZ16	0	14	3.11	94.02
	MY16	0	13.3	3.03	
	CZ17	0	13.02	2.11	
	MY17	0	6.02	0.90	
	CZ18	0	12.46	2.31	
	BLUP	0.28	9.47	2.27	

^a^ infection type

^b^ final disease severity

^c^ the area under disease progress curve

### Genetic diversity analysis

After filtering, 6404 polymorphic markers (comprising 5898 polymorphic DArT-seq markers and 506 polymorphic allele variations for SSR markers) were retained for the 143 accessions. Among these markers, 2120, 3229 and 1055 markers were located in the A, B and D subgenomes, respectively. Chromosome 2B (709) carried the most markers, whereas chromosome 4D (52) carried the fewest markers. Gene diversity, polymorphism information content (PIC) and minor allele frequency (MAF) for the entire genome ranged from 0.2879 to 0.3653, 0.2355 to 0.2916 and 0.2070 to 0.2800 with averages of 0.3288, 0.2664 and 0.2390, respectively. Subgenome B showed the highest gene diversity, PIC and MAF values (0.3307, 0.2674 and 0.2407, respectively). Subgenome D exhibited the lowest gene diversity, PIC and MAF values (0.3232, 0.2630 and 0.2319, respectively). Among individual chromosomes, chromosome 6A carried 376 markers and showed the highest genetic diversity, PIC and MAF values, whereas chromosome 2D carried 270 markers and exhibited the lowest genetic diversity, PIC and MAF values (Table 2).

**Table 2:** Summary of genetic diversity of 143 wheat accessions on sub-genomes and chromosomes

Chromosome	Number of markers	PIC ^a^	Gene Diversity	Minor Allele Frequency
1A	265	0.2603	0.3188	0.2260
2A	485	0.2875	0.3620	0.2800
3A	241	0.2605	0.3203	0.2315
4A	344	0.2696	0.3332	0.2435
5A	134	0.2634	0.3258	0.2403
6A	376	0.2916	0.3653	0.2755
7A	275	0.2580	0.3164	0.2265
**A genome**	**2120**	**0.2687**	**0.3324**	**0.2443**
1B	540	0.2777	0.3456	0.2540
2B	709	0.2741	0.3418	0.2570
3B	642	0.2649	0.3272	0.2381
4B	192	0.2647	0.3269	0.2349
5B	521	0.2487	0.3028	0.2123
6B	341	0.2638	0.3245	0.2323
7B	284	0.2782	0.3463	0.2563
**B genome**	**3229**	**0.2674**	**0.3307**	**0.2407**
1D	125	0.2631	0.3219	0.2267
2D	270	0.2355	0.2879	0.2070
3D	144	0.2589	0.3162	0.2188
4D	52	0.2828	0.3492	0.2513
5D	112	0.2547	0.3126	0.2277
6D	161	0.2807	0.3497	0.2644
7D	191	0.2652	0.3251	0.2274
**D genome**	**1055**	**0.2630**	**0.3232**	**0.2319**
**Whole genome**	**6404**	**0.2664**	**0.3288**	**0.2390**

^a^ polymorphism information content

### Population structure, kinship and LD analyses

The population structure (Q-matrix) was calculated by means of Bayesian clustering using the 6404 polymorphic markers for the 143 accessions, which were divided into two subgroups, designated subgroup 1 (Gp1) and subgroup 2 (Gp2) (Additional file 3a). Gp1 contained 67 accessions, which originated from Sichuan (52), Yunnan (7), Shaanxi (5), Gansu (2) and Guizhou (1) provinces. Gp2 consisted of 76 accessions that originated from Fujian (6), Gansu (5), Guangdong (12), Guangxi (4), Guizhou (14), Hunan (1), Jiangxi (1), Shaanxi (1), Sichuan (18) and Yunnan (14) provinces. On the basis of IT scores, Gp1 contained a higher number of accessions (33) that showed resistance to stripe rust than that of Gp2 (12) in all five environments (Additional file 1). All accessions in each subgroup (Gp1 and Gp2) formed a single cluster (Additional file 3b). The extent of LD and average rate of LD decay of the 143 genotypes was graphically displayed based on pairwise LD squared correlation coefficients (*r*^2^) for all intra-chromosomal markers against the genetic distance (Additional file 4). The half-decay distance was 4 cM when the LD declined to 50% (*r*^2^ = 0.25) of its initial value. Hence, the significant associated loci on the same chromosome within the confidence interval of ±4 cM were considered to be located in the same quantitative trait locus (QTL) block.

### Marker–trait associations at the seedling stage

**Fig. 2:**
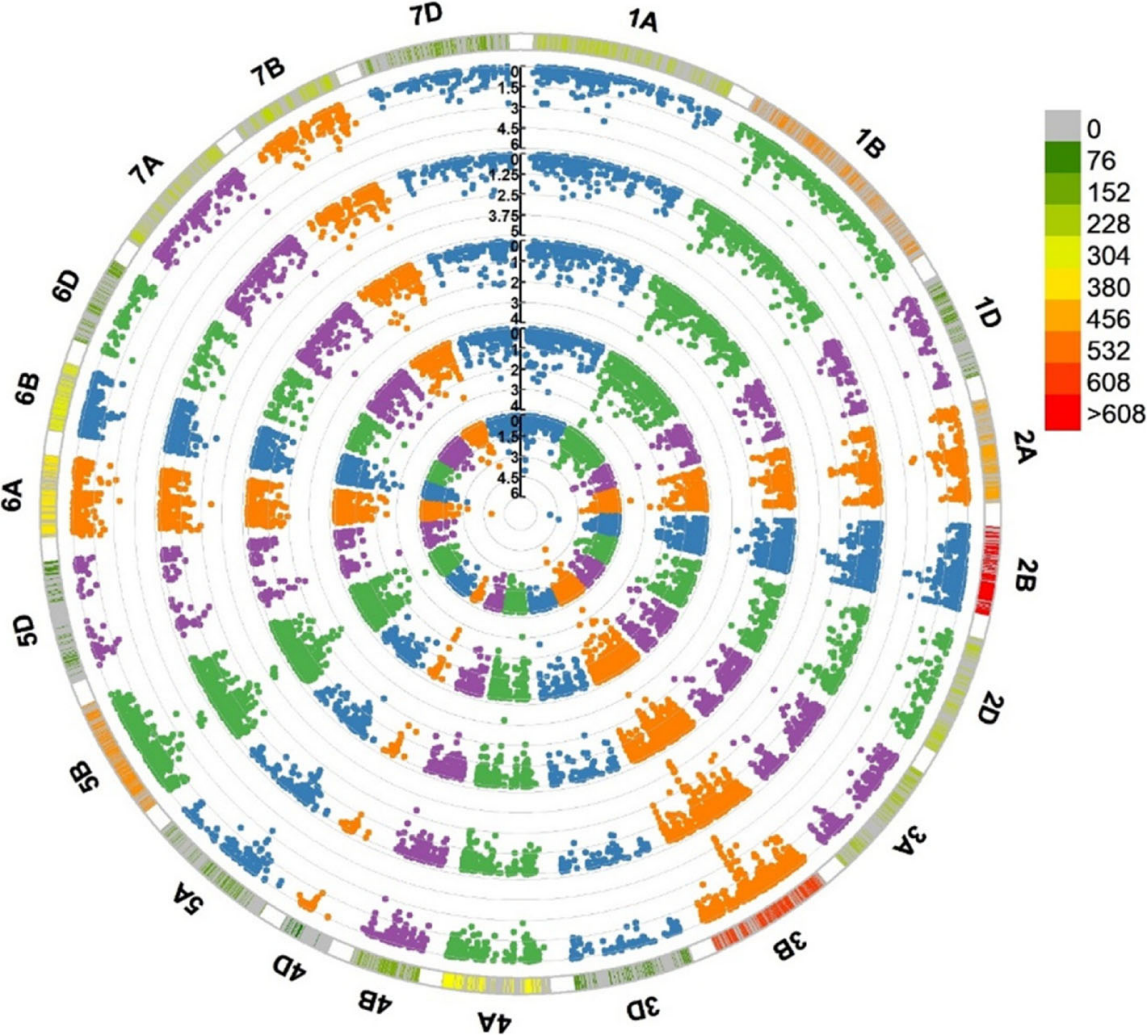
The MLM Manhattan plot of stripe rust resistance significantly associated markers. The horizontal line shows the genome-wide significant threshold –log10(*P*) value of 3.0. The associated MTAs for IT of CYR32, CYR34 with seedling resistance, IT, FDS and AUDPC based on the BLUP from the inner circle to the outer circle

**Fig. 3:**
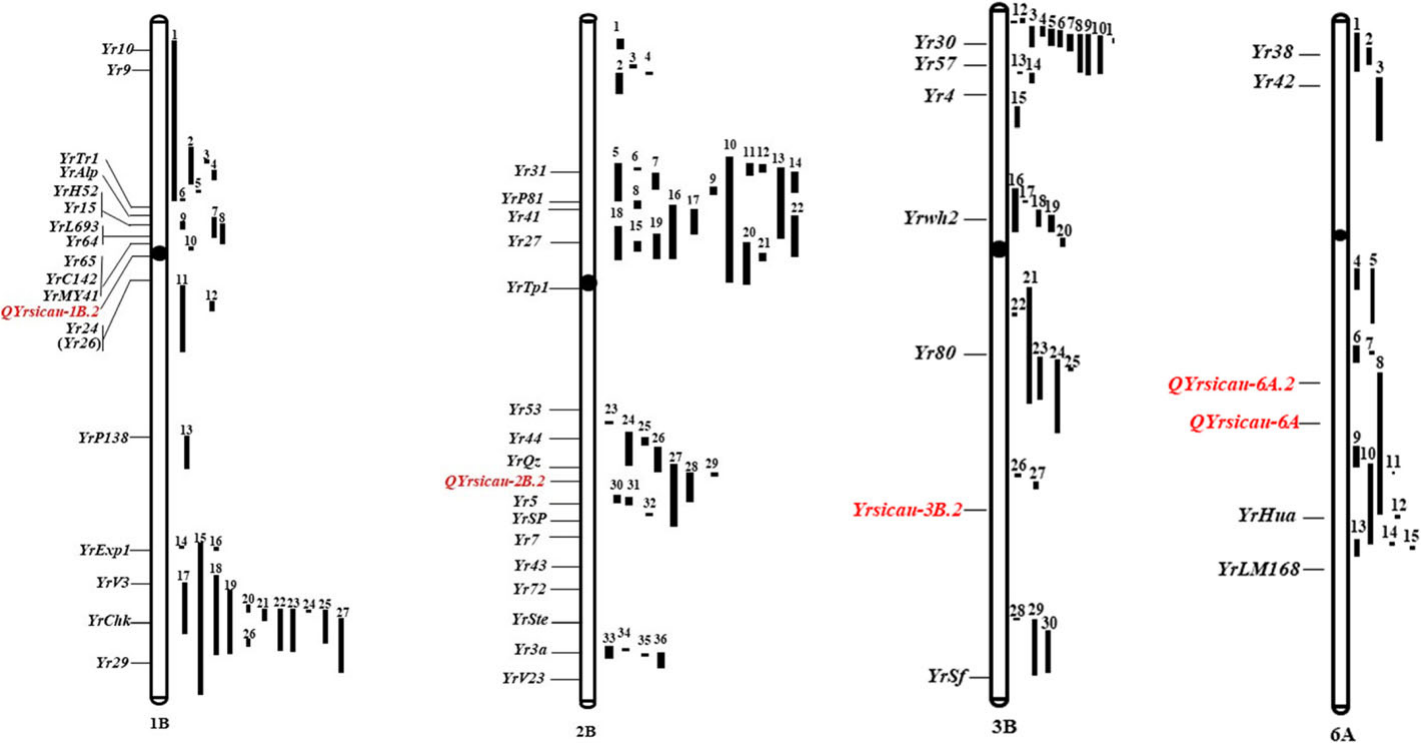
The position of the potentially novel QTL on chromosomes 1B, 2B, 3B and 6A in this study. QTL marked as red color on the left side of chromosomes were the potentially new QTL in this study. The reported genes and QTL were marked as black color and mapped on the left and right side of the chromosomes separately

Using data for the 6404 polymorphic markers, a GWAS analysis was performed for stripe rust IT to a single *Pst* race (CYR32 or CYR34) at the seedling stage based on a mixed linear model. The GWAS for IT identified a total of 18 DArT-seq markers and one SSR marker within 17 QTL on 11 chromosomes as significantly associated (*P* < 0.001) with seedling resistance; these markers were located on chromosomes 1A, 1B, 2A, 2B, 3B, 4A, 5B, 6A, 6B, 7B and 7D (Fig. 2). The phenotypic variation explained (PVE) by the marker–trait associations ranged from 8.71 to 17.94% (Table 3). Based on the LD decay distance observed in this study, significant markers within 4 cM were combined as a QTL, hence 17 QTL regions were detected with IT. Of these QTL, 10 QTL were significantly associated with ASR to CYR32 and seven QTL were significantly associated with ASR to CYR34. Thirteen of these QTL corresponded with previously reported genes or QTL, and four potentially novel QTL associated with seedling resistance were identified on chromosomes 1B, 2B, 3B and 6A (Fig. 3, Additional file 5).

**Table 3:** The summary of QTL and significant markers associated with stripe rust seedling response for CYR32 and CYR34 in the panel

QTL Name	Races	Trait	Marker	Chromosome	Position (cM)	Position (Mb)	−log 10 (*P*)	Marker *R*^**2**^ (%)	References
*Yrsicau-1A*	CYR32	IT	*1,279,571*	1A	39.29	32.54	3.24	11.14	[38]
	CYR32	IT	*1,067,220*	1A	42.17	24.57	4.01	13.96	
*Yrsicau-2B.1*	CYR32	IT	*1,055,456*	2B	0.98	8.50	5.03	17.81	[39, 41]
*Yrsicau-2B.2*	CYR32	IT	*1,687,674*	2B	74.14	273.69	4.36	15.28	
*Yrsicau-3B.1*	CYR32	IT	*4,989,942*	3B	53.54	331.90	4	13.91	[20]
*Yrsicau-3B.2*	CYR32	IT	*3,953,802*	3B	116.07	772.47	3.12	10.7	
*Yrsicau-6A.1*	CYR32	IT	*1,721,876*	6A	29.3	19.04	5.07	17.94	[42]
*Yrsicau-6A.2*	CYR32	IT	*1,103,920*	6A	84.01	595.67	3.3	11.36	
*Yrsicau-6B.1*	CYR32	IT	*3,533,808*	6B	24.83	62.53	3.18	10.93	[30, 31, 43–46]
*Yrsicau-7B*	CYR32	IT	*1,121,184*	7B	129.77	745.04	3.41	11.74	[47, 48]
*Yrsicau-7D*	CYR32	IT	*Xgwm111*	7D		13.46	3.22	8.71	[30]
*Yrsicau-1B.1*	CYR34	IT	*5,325,193*	1B	50.15	29.51	3.83	13.3	[38, 49]
	CYR34	IT	*1,261,119*	1B	51.29	326.93	3.61	12.5	
*Yrsicau-1B.2*	CYR34	IT	*1,094,760*	1B	111.34	448.74	3.08	10.56	
*Yrsicau-2A*	CYR34	IT	*993,667*	2A	73.88	602.69	3.67	12.7	[30, 38]
*Yrsicau-3B.3*	CYR34	IT	*1,143,801*	3B	70.64	636.44	3.5	12.07	[50]
*Yrsicau-4A*	CYR34	IT	*2,288,912*	4A	29.37	583.02	3.04	10.43	[31, 39]
*Yrsicau-5B*	CYR34	IT	*4,408,847*	5B	68.21	546.83	3.59	12.43	[30, 31, 36]
*Yrsicau-6B.2*	CYR34	IT	*1,206,552*	6B	31.49	378.40	3.08	10.55	[31, 51]

### Marker–trait associations at the adult-plant stage

Following the same procedure, the GWAS analysis was also performed for IT, FDS and AUDPC of stripe rust against the mixed *Pst* races within five environments at the adult-plant stage. A total of 32 markers (31 DArT-seq markers and one SSR marker) within 15 QTL on seven chromosomes were identified as significantly associated (*P* < 0.001) with APR in at least two environments; these markers were located on chromosomes 1B, 2A, 2B, 3B, 4A, 5B and 6A (Fig. 2). The PVE by the marker–trait associations ranged from 8.09 to 23.77% (Table 4). On chromosomes 1B, 2B and 4A, five markers were associated with one trait (IT, FDS, or AUDPC). In addition, 27 markers represented loci significantly associated with stripe rust FDS and AUDPC on chromosomes 1B, 2A, 2B, 3B, 5B and 6A. The ranges in PVE for the FDS and AUDPC loci were in the ranges 8.09–20.92% and 8.16–23.77%, respectively. Based on the LD decay distance observed in this study, significant markers within 4 cM were combined as a QTL, hence a total of 15 QTL regions for IT, FDS, and AUDPC were detected. Chromosome 1B contained four QTL, chromosomes 3B and 5B carried three QTL each, chromosome 2B included two QTL and one QTL was detected on each of chromosomes 2A, 4A and 6A. Among these QTL, 11 QTL linked to one marker were associated with IT, FDS, or AUDPC, respectively. The locus *QYrsicau-5B.3* linked to *1,108,002* and *1,223,817* was associated with both FDS and AUDPC and the PVE was 13.75–20.08% and 14.39–23.3%, respectively. *QYrsicau-2B.1* and *QYrsicau-5B.2* were linked to three and six markers, respectively. Notably, *QYrsicau-3B.3* was linked to ten markers, of which *1,129,542* was associated with both FDS and AUDPC in three and five environments and the PVE was 19.66 and 19.29%, respectively. Fourteen QTL corresponded with previously reported genes or QTL. *QYrsicau-6A* was a potentially novel QTL associated with the adult-plant stage response (Fig. 3, Additional file 5). Notably, four QTL (*QYrsicau-1B.2*, *QYrsicau-2B.1*, *QYrsicau-3B.2* and *QYrsicau-5B.3*) on chromosomes 1B, 2B, 3B and 5B were detected at the seedling and adult-plant stages for which the PVE ranged from 9.99 to 23.30%, respectively.

**Table 4:** The summary of QTL for stripe rust resistance identified at the adult plant stage across five experiments in the panel

QTL Name	Marker	Chromosome	Position (cM)	Position (Mb)	Trait	Environment	−log 10 (*P*)	Marker *R*^2^ (%)	References
*QYrsicau-1B.1*	*1,255,154*	1B	32.28	13.09	AUDPC	CZ16, MY16, MY17	3.11–3.92	10.31–13.21	[20, 30, 49, 52]
*QYrsicau-1B.2*	*4,537,457*	1B	51.29	3.16	FDS	CZ17	4.44	15.04	[20, 31, 39, 53]
					AUDPC	CZ16, MY16, CZ17, BLUP	3.36–3.62	11.09–12.27	
*QYrsicau-1B.3*	*Xgwm268*	1B		637.37	AUDPC	CZ16, MY16	3.55–4.48	9.4–12.58	[54, 55]
*QYrsicau-1B.4*	*1,161,065*	1B	286.65	681.08	FDS	CZ17, MY17	3.53–4.91	9.39–14.14	[56]
					AUDPC	CZ17, MY17, BLUP	3.12–5.01	8.23–14.45	
*QYrsicau-2A*	*4,004,515*	2A	60.91	72.69	FDS	MY16, MY17	3.41–5.65	11.33–19.68	[30, 39, 57–59]
					AUDPC	MY16, MY17, BLUP	3.69–5.05	11.91–17.35	
*QYrsicau-2B.1*	*1,263,973*	2B	71.82	184.66	FDS	CZ16, CZ18	3.11–3.38	10.62–11.26	[41, 60–62]
	*1,138,058*	2B	73.02	235.16	FDS	MY17	3.22	10.77	
					AUDPC	CZ16, BLUP	3.02–3.32	9.99–10.64	
	*4,663,985*	2B	74.08	383.85	FDS	CZ17, MY17	3.27–3.29	10.92–10.93	
					AUDPC	MY16, CZ17, BLUP	3.2–3.96	10.76–12.85	
*QYrsicau-2B.2*	*1,254,647*	2B	107.03	798.29	AUDPC	CZ16, CZ17, MY16, BLUP	3.05–3.98	9.99–13.39	[20, 47, 63]
*QYrsicau-3B.1*	*3,943,894*	3B	20.8	25.29	FDS	MY16	3.21	8.34	[62, 64, 65]
					AUDPC	CZ17, MY16, MY17	3.16–4.08	8.25–11.14	
*QYrsicau-3B.2*	*1,133,063*	3B	68.59	612.30	FDS	CZ17, MY17	3.11–3.33	10.28–11.15	[41, 50]
					AUDPC	CZ16, CZ17, BLUP	3.18–4.61	10.57–15.88	
*QYrsicau-3B.3*	*1,086,466*	3B	90.44	739.04	FDS	MY17, BLUP	3.66–5.65	11.94–19.67	[66, 67]
					AUDPC	MY17, BLUP	4.39–5.57	14.34–19.31	
	*1,244,635*	3B	90.68	742.26	FDS	MY17, BLUP	4.01–5.66	13.16–19.72	
					AUDPC	CZ17, MY17, BLUP	3.11–5.6	10.46–19.41	
	*1,129,542*	3B	90.68	740.11	FDS	CZ17, MY17, BLUP	3.12–6.45	8.09–19.66	
					AUDPC	CZ16, CZ17, MY16, MY17, BLUP	3.16–6.37	8.16–19.29	
	*2,275,715*	3B	90.68	742.17	FDS	CZ17, MY17, BLUP	3.43–5.66	11.42–19.71	
					AUDPC	CZ17, MY16, MY17, BLUP	3.065.9	10.04–20.58	
	*1,102,869*	3B	91.03	741.30	FDS	MY17, BLUP	3.81–5.65	12.44–19.68	
					AUDPC	MY16, MY17, BLUP	3.56–5.61	11.77–19.47	
	*2,279,272*	3B	91.04	739.04	FDS	MY17, BLUP	4.32–5.9	14.23–20.65	
					AUDPC	CZ17, MY16, MY17, BLUP	3.13–5.82	10.54–20.26	
	*1,138,233*	3B	92.78	744.32	FDS	MY17, BLUP	3.09–4.73	9.97–16.2	
					AUDPC	MY17, BLUP	3.56–4.94	11.47–16.93	
	*1,107,260*	3B	93.62	740.11	FDS	MY17, BLUP	3.08–3.65	9.95–12.28	
					AUDPC	CZ16, MY17, BLUP	3.04–4.1	10.07–13.87	
	*3,940,970*	3B	92.68	741.50	FDS	MY17, BLUP	3.63–5.97	11.83–20.92	
					AUDPC	CZ17, MY17, BLUP	3.09–5.66	10.39–19.64	
	*4,439,724*	3B	92.68	743.51	FDS	MY17, BLUP	4.16–5.19	13.69–17.91	
					AUDPC	MY17, BLUP	4.34–5.43	14.17–18.8	
*QYrsicau-4A*	*1,231,042*	4A	83.92		IT	CZ16, CZ17, BLUP	3.13–3.36	10.41–11.31	[59]
*QYrsicau-5B.1*	*3,944,166*	5B	50.14	511.71	FDS	CZ17, MY17, BLUP	3.99–5.47	13.08–18.86	[30, 68]
					AUDPC	CZ17, MY16, MY17, BLUP	3.99–6.07	13.28–21.23	
*QYrsicau-5B.2*	*3,022,447*	5B	55.6	503.08	FDS	CZ17, MY16, MY17, BLUP	3.1–4.82	10.25–16.53	[66, 69]
					AUDPC	MY16, MY17, BLUP	4.87–6.63	16.03–23.41	
	*1,103,656*	5B	55.6	506.96	FDS	CZ17, MY16, MY17, BLUP	3.4–5.75	11.27–19.88	
					AUDPC	CZ17, MY16, MY17, BLUP	4.27–5.99	14.31–20.92	
	*3,936,865*	5B	55.6	527.15	FDS	CZ17	4.05	13.61	
					AUDPC	CZ17, MY17, BLUP	3.11–5.32	9.94–18.57	
	*3,024,339*	5B	55.71	527.03	FDS	MY16, MY17, BLUP	3.47–4.97	11.53–17.12	
					AUDPC	MY16, MY17, BLUP	5.13–6.72	16.95–23.77	
	*2,276,711*	5B	57.24	522.95	FDS	CZ17, MY17, BLUP	3.98–5.47	13.06–18.85	
					AUDPC	CZ17, MY16, MY17, BLUP	4.01–5.99	13.36–20.91	
	*3,956,366*	5B	59.68	511.61	FDS	CZ17, MY17	3.07–4.17	10.23–14.06	
					AUDPC	CZ17, MY17	3.92–4.25	13.35–14.42	
*QYrsicau-5B.3*	*1,108,002*	5B	64.83	510.88	FDS	CZ17, MY17, BLUP	4.18–5.75	13.75–20.05	[31, 52, 70]
					AUDPC	CZ17, MY16, MY17, BLUP	4.3–6.6	14.39–23.3	
	*1,223,817*	5B	66.35	523.93	FDS	CZ17, MY17, BLUP	4.26–5.8	14.04–20.08	
					AUDPC	CZ17, MY16, MY17, BLUP	4.31–6.09	14.44–21.31	
*QYrsicau-6A*	*3,021,470*	6A	78.71	609.38	FDS	CZ17	3.44	11.44	
					AUDPC	CZ16, CZ17, BLUP	3.06–4.2	9.76–14.38	

### Favorable allele analyses

**Fig. 4:**
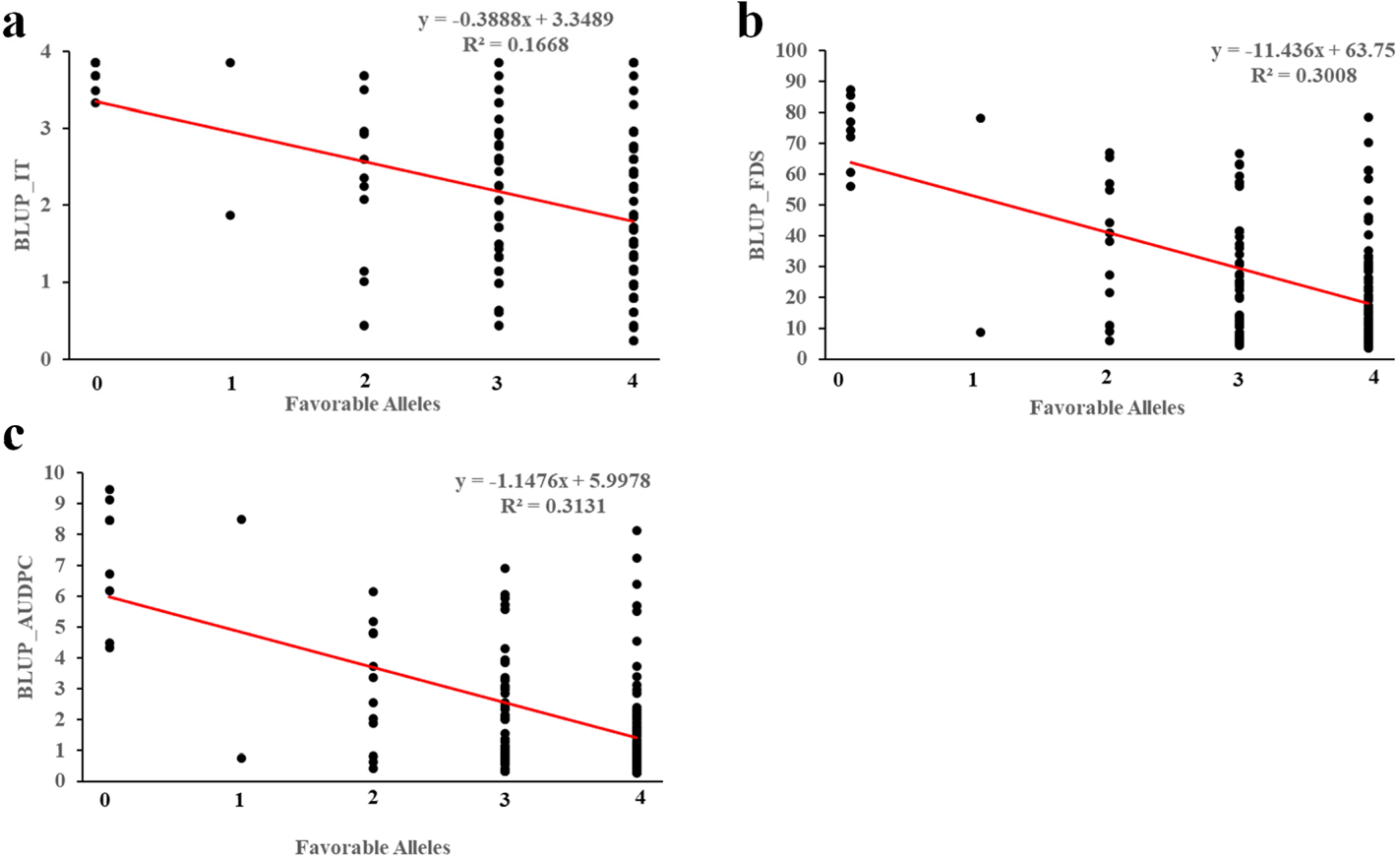
Regression of reaction to *Pst* against number of favorable alleles in 143 wheat accessions. (**a**) BLUP_IT (**b**) BLUP_FDS (**c**) BLUP_AUDPC. Four stable QTL for APR, including *QYrsicau-2B.1*, *QYrsicau-3B.3*, *QYrsicau-5B.2* and *QYrsicau-5B.3*, were selected for analysis

Four QTL were significantly associated with stripe rust in at least four environments in the field. These stable QTL, consisting of *QYrsicau-2B.1*, *QYrsicau-3B.3*, *QYrsicau-5B.2* and *QYrsicau-5B.3*, showed the highest frequencies (68.53–86.71%) among the favorable resistance-associated alleles in the 143 accessions. We investigated the additive effects of the favorable alleles of these four APR QTL on the traits BLUP_IT, BLUP_FDS and BLUP_AUDPC (Fig. 4). A significant negative correlation was identified between the number of favorable alleles in individual accessions and the respective stripe rust IT, FDS and AUDPC, with *R*^2^ values of 0.17, 0.30 and 0.31, respectively. These results indicated that accessions with favorable alleles exhibited higher resistance to stripe rust, and supported the use of a combination of several loci for wheat disease-resistance breeding (Fig. 4).

## Discussion

### Stripe rust resistance in the wheat landrace diversity panel from the southern autumn-sown spring wheat zone of China

In this study, 143 common wheat landrace accessions from the southern autumn-sown spring wheat zone of China were evaluated for resistance against *Pst* at the seedling and adult-plant stages. Based on IT scores, 33 (49.25%) resistant accessions in this panel were clustered in Gp1, whereas Gp2 contained 12 (15.79%) accessions. Interestingly, all of these 45 accessions originated from southwestern provinces, namely Sichuan (26 accessions), Yunnan (8), Shaanxi (4), Guizhou (4) and Gansu (3). China is considered to be a unique epidemiological zone [1]. The autumn-sown spring wheat production areas of these provinces are located within stripe rust epidemic regions in China [23, 26]. In particular, southern Gansu and northwestern Sichuan comprise a “center of origin for virulence” [8]. Understandably, resistant accessions were more likely to be selected by farmers among wheat landraces grown in the stripe rust epidemic regions. Furthermore, a majority of resistant accessions in this panel displayed APR resistance to stripe rust, suggesting that race non-specific and durable resistance genes might be favored by artificial selection in Chinese wheat landraces to provide durable resistance. For example, ‘Chinese Spring’, which is a wheat landrace originating from Sichuan province, showed stable resistance to stripe rust across all environments at the adult-plant stage. This accession carries *Yr18* [71], which is a durable stripe rust resistance gene that is frequently present in Chinese wheat landraces [72]. Such resistant accessions from Chinese wheat landraces represent a valuable resource for development of durable stripe rust resistant cultivars in wheat breeding.

### Comparison of high-confidence loci with adult-plant resistance other wheat zones of China

Thirty-two markers linked with 15 QTL on seven chromosomes were identified as significantly associated (*P* < 0.001) with IT, FDS or AUDPC in at least two environments with APR. Six putative QTL for stripe rust resistance have been identified previously in Chinese landrace wheat populations from different wheat-growing zones [20, 30, 31]. Five of these QTL, including *QYrsicau-1B.1*, *QYrsicau-1B.2*, *QYrsicau-2A*, *QYrsicau-5B.1* and *QYrsicau-5B.3*, were located close to QTL previously identified in accessions from the Yellow and Huai River Valleys [30]. *QYrsicau-1B.1*, *QYrsicau-1B.2* and *QYr.sicau-2B.2* were located close to QTL previously identified in landraces from the middle and lower reaches of the Yangtze River [20]. Only two QTL, *QYrsicau-1B.2* and *QYrsicau-5B.1*, were identified in the northern Chinese wheat zone [31]. The QTL shared among wheat zones likely originated in ancestral landraces and the present-day distribution of these QTL might reflect the historical spread of wheat in China [73] and differences in selection pressures for stripe rust. Nine QTL were unique to the southern autumn-sown spring wheat zone of China, suggesting that wheat landraces from this zone harbor unique characteristics in the genetic diversity of resistance to stripe rust and may be used as novel germplasm resources for stripe rust resistance breeding.

### Novel stripe rust resistance loci

In the present landrace wheat panel, 19 loci within 17 QTL were significantly associated with ASR to *Pst* detected in the seedling test. However, no overlap in QTL for seedling resistance to the two races CYR32 and CYR34 was observed, presumably because few accessions were resistant to both *Pst* races in this panel. Of these QTL, four QTL differed from previously identified genes or QTL for resistance to *Pst* (Table 3). Three potentially novel loci (*Yrsicau-2B.2*, *Yrsicau-3B.2*, and *Yrsicau-6A.2*) were associated with resistance to CYR32, and *Yrsicau-1B.2* was associated with resistance to CYR34. *Yrsicau-1B.2* was closely associated with *YrC142*, which is a temporarily designated stripe rust resistance gene in synthetic wheat CI142 [74]. However, CI142 is a synthetic wheat line originating from a durum wheat (*Triticum durum*) × *Aegilops tauschii* cross. There is a negligible likelihood that a QTL in a Chinese wheat landrace is identical to one that originated in durum wheat. *Yrsicau-2B.2* was located close to *QYraq.cau-2BL* flanked by the microsatellite markers *Xwmc175* and *Xwmc332*. *QYraq.cau-2BL* is derived from an Italian winter wheat cultivar Aquileja [75] and is an APR locus. Thus, the ASR locus *Yrsicau-2B.2* is predicted to differ from *QYraq.cau-2BL*. Based on the consensus map, *Yrsicau-3B.2* identified by the marker *3,953,802* and *Yrsicau-6A.2* identified by *3,021,470* are unlikely to be closely linked with previously identified genes or QTL. Therefore, these four ASR loci are potentially novel. Several accessions that show ASR to stripe rust were observed to carry these novel loci. For example, Yuqiumai (AS661657), Zhenixiaomai (AS661777) and Guangtoumai (AS661671), which show resistance to both CYR32 and CYR34, carried the resistance alleles of *Yrsicau-1B.2* and *Yrsicau-3B.2.* These resistant accessions carrying novel ASR loci could be utilized for development of wheat cultivars possessing ASR to stripe rust.

In addition, 32 markers within 15 QTL on seven chromosomes were identified as significantly associated (*P* < 0.001) with IT, FDS or AUDPC in at least two environments with APR (Table 4). However, all of these QTL except *QYrsicau-6A* were tightly linked or overlapped with the positions of known APR genes or QTL (Table 4). *QYrsicau-6A* was identified by the DArT-seq marker *3,021,470*, which was located on the long arm of chromosome 6A at ~ 609.4 Mb and explained 9.76–14.38% of the phenotypic variation across different environments. This novel QTL was detected in 13 accessions that showed high levels of APR for stripe rust (IT ≤1) (Additional file 1). These resistant accessions may serve as favorable donor parents of APR for wheat breeding.

## Conclusions

In this study, we evaluated the stripe rust resistance of 143 wheat landrace accessions from the southern autumn-sown spring wheat zone of China. Seventeen accessions showed stable high-level resistance to stripe rust at the adult-plant stage in five test environments, whereas four accessions showed resistance to the *Pst* races CYR32 and CYR34 at the seedling stage. The GWAS results revealed that 19 loci within 17 QTL were significantly associated with ASR, and 32 loci within 15 QTL were identified as significantly associated with APR. Among these loci were five potentially novel QTL. The identified resistant accessions and resistance loci will be useful in the ongoing effort to develop new wheat cultivars with strong resistance to stripe rust.

## Methods

### Plant materials

A collection of 143 common wheat Chinese landrace accessions obtained from the Chinese Academy of Agricultural Sciences of National Germplasm Repository were used in this study. These accessions were originated from 10 Chinese provinces, namely Sichuan (70), Yunnan (21), Guizhou (15), Guangdong (12), Gansu (7), Fujian (6), Shaanxi (6), Guangxi (4), Hunan (1) and Jiangxi (1). The list of accessions is provided in Additional file 1.

### Greenhouse evaluation

Evaluation of the IT response of wheat seedlings to two prevalent Chinese *Pst* races (CYR32 and CYR34) was performed under a controlled greenhouse environment at the Plant Protection Institute of the Gansu Academy of Agricultural Sciences, Gansu, China. The avirulence/virulence classification of the *Pst* races is provided in Additional file 6 [9, 24, 37, 49, 76–80]. Five to six seeds of each accession were sown in a plastic pot filled with nutrient soil. Seedlings of each accession were inoculated with *Pst* races when plants were at approximately the two-leaf stage. First, a spore suspension (fresh uredospores:aqueous Twain, 25:1, m/V) was prepared. The spore suspension was evenly sprayed on the leaves of the plants. The suspension was left for 30 min to dry. The inoculated plants were placed in a dark dew chamber in full humidity for 24 h at 10–15 °C. Subsequently, the plants were moved to a greenhouse maintained at 15–16 °C. A photoperiod of 12–14 h light and 10–12 h darkness was maintained throughout the experiment. The susceptible control was the highly susceptible wheat cultivar Mingxian 169. The IT was scored 15–18 d after inoculation [81] using the 0–4 scale described previously, as follows: resistant (0–2) and susceptible (3–4) [82].

### Field evaluation

All accessions were assessed for stripe rust resistance at the adult-plant stage after artificial inoculation in 5 year-location environments performed at two field sites in Sichuan Province, namely Chongzhou (CZ; 30°33′N, 103°39′E) and Mianyang (MY; 31°23′N, 104°49′E). Seeds were sown at Chongzhou in late October and at Mianyang in early November. The evaluations were performed at Chongzhou from 2016 to 2018 (three crop seasons) and at Mianyang in 2016 and 2017 (two crop seasons), which were designated CZ16, CZ17, CZ18, MY16 and MY17, respectively.

In all field trials, five randomly chosen plants of all accessions were evaluated per three replicate rows. Plots were prepared as 1.50-m-long rows, spaced 0.30 m apart, and sown with 15 seeds for each accession. Two highly susceptible common wheat cultivars, SY95–71 and Taichung 29, used as a spreader border were planted around each plot and every 20 rows. At the tillering stage, an equal number of mixed *Pst* races and talc (1:50, m/V) was mixed evenly, and the daubing method was used for artificial inoculation. Plants were inoculated with a mixture of Chinese prevalent *Pst* races (CYR 32, CYR 33, CYR 34, Sull-4, Sull-5, Sull-7 and G22–14).

Stripe rust responses were recorded when the susceptible cultivars SY95–71 and Taichung 29 displayed disease severity (DS) of up to 80%. In all trials, stripe rust resistance was evaluated three times at weekly intervals. We scored IT using the 0–4 scale described previously [82]. The DS was scored as percentage of infected leaf area (0, 5, 10, 20, 40, 60, 80% or 100%) in accordance with the standard for monitoring and forecasting wheat stripe rust (National Standard of the People’s Republic of China, GB/T 15795–2011). Data for final disease severity (FDS) were used for GWAS analysis. The DS was used to calculate the AUDPC using the following formula: AUDPC = ${\sum}_{i=1}^{n-1}\left[\left({x}_{i+1}+{x}_i\right)/2\right]\left({t}_{i+1}-{t}_i\right)$, where *x*_*i*_ = flag leaf rust severity on the *i*th date, *t*_*i*_ = the *i*th day and *n* = number of times on which DS was recorded [83].

### Phenotypic data analysis

To eliminate the impact of environmental factors on stripe rust responses, BLUP values for each accession across environments were calculated by a linear model with random effects for variance components using the lme4 package in R [84]. The broad-sense heritability (*H*^2^) estimates for IT, FDS and AUDPC were calculated for each environment using QTL IciMapping v4.1 [85] with the formula *H*^2^ = *V*_G_/(*V*_G_ + *V*_E_), where *V*_G_ and *V*_E_ are estimates of the genetic and environmental variances, respectively [86]. A Pearson’s correlation analysis of BLUP values for the five environments was performed using IBM SPSS Statistics 20.0 (IBM Corp., Armonk, NY, USA). The phenotypic variation was estimated as the minimum, maximum and mean values of all traits in the five environments and BLUP values.

### Genotyping and genetic diversity

Genomic DNA was extracted from fresh leaf tissue from each accession using the modified cetyltrimethylammonium bromide method [87]. DNA samples were diluted to a working solution of 50–100 ng/μL with an *A*_260_/ *A*_280_ ratio of 1.8–2.0. The panel of 143 wheat landraces was used for genotyping based on DArT-seq technology (Diversity Arrays Technology, Canberra, ACT, Australia). A total of 133 SSR markers, associated with stripe rust resistance genes, were obtained from the GrainGenes database (http://wheat.pw.usda.gov) and previous reports [88–91], and used for additional genotyping. All SSR markers were subjected to PCR amplification in a reaction volume of 3 μL. The PCR products were separated by 6% denaturing polyacrylamide gel and visualized by silver staining [92]. For quality control, markers with missing values > 10% and MAF < 5% were removed [93]. After applying these filtering criteria, 5898 DArT-seq markers and 133 SSR markers with 506 polymorphic allele variations were used to estimate population structure and kinship coefficients for the GWAS. The PIC values were calculated for each marker using the formula PIC = 1 − ∑(*P*_*i*_)^2^, where *P*_*i*_ is the proportion of the population carrying the *i*th allele [94]. PowerMarker v3.25 [95] was used to estimate PIC, MAF and gene diversity of the DArT-seq and SSR data.

### Population structure, kinship and LD analysis

A population structure analysis was performed using the Bayesian clustering algorithm implemented in STRUCTURE v2.3.4 [96]. The data set comprised 6404 markers, including 5898 DArT-seq and 506 polymorphic allele variations from SSR markers. In total, ten independent STRUCTURE runs were performed with *K-*value varying from 1 to 10 using the admixture model with 10,000 replicates for burn-in and 10,000 replicates for Markov chain Monte Carlo iterations [93]. The optimal *K-*value was determined using the delta *K* method [97]. Kinship among the 143 wheat landrace accessions was estimated with the 6404 markers using TASSEL v3.0. The LD across the known genetic distance for each chromosome of all accessions was calculated using TASSEL v3.0 [98] with 5898 DArT-seq markers. The LD squared allele frequency correlation was evaluated for the entire genome. Significant pair-wise markers were chosen using the criteria *P* < 0.001 and *r*^2^ > 0.1. The LD decay plot and half-decay distance were generated using *r*^2^ and the genetic map distance between markers. All high-confidence associated loci in the half decay distance region on the same chromosome were combined as a single QTL.

### Association analysis

To identify loci associated with the response of the 143 accessions to *Pst* races, GWAS analyses were performed using 6404 markers and the mixed linear model with Q and K as covariates implemented in TASSEL v3.0 software [99]. Association tests were conducted for phenotypic traits values (IT, FDS and AUDPC) from all single environments and the BLUP values. The significance threshold was −log_10_(*P*) > 3 [100]. Significant markers were visualized with a Manhattan plot using the “Manhattan” function in the “qqman” package [99] in R × 64 3.6.3. The loci that showed a significant association detected in at least two environments were selected for further analyses.

### Comparison of QTL locations with previously reported *Yr* genes and QTL

We compared the locations of significant QTL determined in this study with those of previously reported *Yr* genes and QTL based on an integrated map to determine whether the QTL were novel. The map included 80 permanently named *Yr* genes, 67 temporarily designated *Yr* genes and 327 previously mapped QTL of DArT-seq, SSR and SNP markers and was generated using BioMercator v4.2 [101, 102]. In the study, physical positions of significant markers were annotated using the reference sequence of bread wheat (IWGSC RefSeq v1.0) [103]. The different markers were combined into a single putative QTL if they were located within a confidence interval of ±4.0 cM (where LD was predicted to fall below the critical threshold of *r*^2^ = 0.3) [104].

## Supplementary Information

**Additional file 1.** 143 wheat landraces used in this study and the infection type (IT) in the seedling stage for CYR32 and CYR34 and IT, final disease severity (FDS) and area under the disease progress curve (AUDPC) in the adult-plant stages among five environments.

**Additional file 2. **Pearson’s correlation coefficients for infection type (IT), final disease severity (FDS) and area under the disease progress curve (AUDPC) against stripe rust evaluated among five environments during 2016 to 2018. Different environments were all correlated, Significant at *P* < 0.01.

**Additional file 3. **Population structure of 143 wheat landrace accessions in Southern Autumn-Sown Spring Wheat Zone of China. (**a**) The population structure of 143 accessions with Bayesian clustering analysis. Two colors stand for 2 different compositions. The Subgroup 1 (Gp1) mainly showed as red color. The Subgroup 2 (Gp2) mainly showed as green color; (**b**) Estimated the distance of hierarchical clustering for the accessions using Fast Ward grouping algorithm and heat map showing the kinship and phylogenetic relations.

**Additional file 4. **Genome-wide average linkage disequilibrium (LD) decay plot for 143 wheat landraces based on 5899 DArT markers. The scatter plots showing pairwise DArT markers LD *r*^2^ value as a function of inter-marker genetic distances (cM).

**Additional file 5. **The position of the potentially novel QTL and details of reported QTL and *Yr* genes located on the integrated map.

**Additional file 6. **The avirulence(A) /virulence(V) formula of the *Pst* races used in this study.

## Data Availability

All the data supporting the results in this article are included in the present and the additional files.

## Abbreviations

APR: Adult-plant resistance
ASR: All-stage resistance
AUDPC: Area under the disease progress curve
BLUP: Best linear unbiased predictor
CYR34: Chinese yellow rust 34
DArT-seq: Diversity Arrays Technology sequencing
DS: Disease severity
FDS: Final disease severity
GWAS: Genome-wide association study
*H*^2^: Broad-sense heritability
IT: Infection types
LD: Linkage disequilibrium
MAF: Minor allele frequencies
MTAs: Marker–trait associations
PIC: Polymorphism information content
*Pst*: *Puccinia striiformis* f. sp. *tritici*
PVE: Phenotypic variation explained
QTL: Quantitative trait locus
SSR: Simple sequence repeat
