# Supplementary material for: Genome-wide association mapping reveals potential novel loci controlling stripe rust resistance in a Chinese wheat landrace diversity panel from the southern autumn-sown spring wheat zone
Source: BMC Genomics. 2021 Jan 7;22:34. doi: 10.1186/s12864-020-07331-1 (PMC7791647; doi:10.1186/s12864-020-07331-1)
Supplement: Supplementary file 1 — Additional file 1. 143 wheat landraces used in this study and the infection type (IT) in the seedling stage for CYR32 and CYR34 and IT, final disease severity (FDS) and area under the disease progress curve (AUDPC) in the adult-plant stages among five environments. [file 12864_2020_7331_MOESM1_ESM.docx]

| **Additional file 1 143 wheat landraces used in this study and the infection type (IT) in the seedling stage for CYR32 and CYR34 and IT, final disease severity (FDS) and area under the disease progress curve (AUDPC) in the adult-plant stages among five environments** | | | | | | | | | | | | | | | | | | | | | | | | | | | | | | | | |  |
| --- | --- | --- | --- | --- | --- | --- | --- | --- | --- | --- | --- | --- | --- | --- | --- | --- | --- | --- | --- | --- | --- | --- | --- | --- | --- | --- | --- | --- | --- | --- | --- | --- | --- |
|  |  |  |  |  |  | Seedling stage | |  | Adult-plant stages | | | | | | | | | | | | | | | | | | | | | | | |  |
| No. | AS_No. ^a^ | Accession Name ^b^ | K=2 | Origin |  | Infection Type (IT) | |  | Infection Type (IT) | | | | | |  | | Disease Severity (DS) (%) | | | | | | |  | | Area under the disease progress curve (AUDPC) | | | | | | |  |
|  |  |  |  |  |  | CYR32 | CYR34 |  | CZ2016 | MY2016 | CZ2017 | MY2017 | CZ2018 | BLUP | |  | | CZ2016 | MY2016 | CZ2017 | MY2017 | CZ2018 | BLUP | |  | | CZ2016 | MY2016 | CZ2017 | MY2017 | CZ2018 | BLUP | |
| 1 | CS | Zhongguochun | 1 | Sichuan |  | 4 | 4 |  | 2 | 1 | 1 | 1 | 1 | 1.32 | |  | | 10 | 5 | 1 | 2 | 6 | 7.79 | |  | | 1.40 | 0.70 | 0.28 | 0.21 | 0.39 | 0.77 | |
| 2 | AS661257 | Lengshanmai | 1 | Shaanxi |  | 4 | 4 |  | 0 | 1 | 0 | 0 | 2 | 0.81 | |  | | 5 | 5 | 0 | 0 | 29 | 11.29 | |  | | 0.18 | 0.70 | 0.28 | 0.00 | 1.51 | 0.77 | |
| 3 | AS661341 | Laomangmai | 2 | Gansu |  | 4 | 4 |  | 3 | 3 | 4 | 1 | 4 | 2.97 | |  | | 60 | 10 | 10 | 1 | 80 | 33.04 | |  | | 3.50 | 1.40 | 1.05 | 0.04 | 4.73 | 2.27 | |
| 4 | AS661545 | Youmanghongke | 2 | Hunan |  | 3 | 3 |  | 4 | 4 | 3 | 4 | 4 | 3.68 | |  | | 80 | 80 | 60 | 52 | 80 | 65.49 | |  | | 3.33 | 6.30 | 6.37 | 2.63 | 6.51 | 4.79 | |
| 5 | AS661549 | Baikeyoumang | 2 | Fujian |  | 3 | 3 |  | 4 | 4 | 4 | 4 | 4 | 3.85 | |  | | 80 | 100 | 44 | 25 | 88 | 63.42 | |  | | 9.80 | 11.20 | 4.55 | 2.10 | 3.75 | 5.74 | |
| 6 | AS661550 | Hongkexiaomai | 2 | Fujian |  | 4 | 4 |  | 4 | 3 | 2 | 3 | 2 | 2.76 | |  | | 80 | 80 | 16 | 25 | 37 | 44.90 | |  | | 5.95 | 6.30 | 2.45 | 1.23 | 2.14 | 3.41 | |
| 7 | AS661552 | Hongkemai | 2 | Fujian |  | 3 | 4 |  | 3 | 3 | 2 | 4 | 3 | 2.95 | |  | | 60 | 80 | 22 | 33 | 25 | 41.66 | |  | | 4.20 | 6.30 | 3.50 | 1.47 | 2.28 | 3.36 | |
| 8 | AS661554 | Youmai | 2 | Guangdong |  | 4 | 3 |  | 4 | 4 | 4 | 4 | 4 | 3.85 | |  | | 80 | 100 | 52 | 38 | 61 | 61.37 | |  | | 9.10 | 10.50 | 5.11 | 1.93 | 4.34 | 5.69 | |
| 9 | AS661555 | Datianquxiaomai | 2 | Guangdong |  | 4 | 4 |  | 4 | 4 | 4 | 4 | 4 | 3.85 | |  | | 100 | 100 | 100 | 80 | 55 | 78.15 | |  | | 14.00 | 11.90 | 13.02 | 5.32 | 3.40 | 8.48 | |
| 10 | AS661557 | Mianmai | 2 | Guangdong |  | 3 | 3 |  | 2 | 2 | 1 | 1 | 4 | 2.08 | |  | | 20 | 20 | 5 | 3 | 48 | 21.55 | |  | | 5.08 | 2.28 | 0.70 | 0.25 | 1.75 | 2.03 | |
| 11 | AS661558 | Qingyuanxiaomai | 2 | Guangdong |  | 3 | 4 |  | 4 | 4 | 3 | 4 | 4 | 3.68 | |  | | 60 | 100 | 60 | 48 | 88 | 66.91 | |  | | 10.50 | 9.10 | 7.00 | 2.52 | 4.48 | 6.14 | |
| 12 | AS661560 | Huoshaomai | 2 | Guangdong |  | 4 | 3 |  | 4 | 3 | 3 | 4 | 4 | 3.50 | |  | | 80 | 40 | 48 | 32 | 100 | 57.08 | |  | | 4.90 | 4.55 | 6.30 | 2.10 | 7.25 | 4.82 | |
| 13 | AS661561 | Kangmai | 2 | Guangdong |  | 4 | 4 |  | 4 | 4 | 3 | 4 | 4 | 3.68 | |  | | 80 | 100 | 44 | 8 | 92 | 61.22 | |  | | 10.50 | 11.20 | 6.16 | 0.56 | 6.30 | 6.41 | |
| 14 | AS661562 | Baikexiaomai | 2 | Guangdong |  | 3 | 3 |  | 3 | 2 | 1 | 3 | 4 | 2.61 | |  | | 60 | 5 | 4 | 11 | 68 | 30.51 | |  | | 7.00 | 0.70 | 0.60 | 0.49 | 2.80 | 2.34 | |
| 15 | AS661563 | Zaoxiaomai | 2 | Guangdong |  | 3 | 4 |  | 4 | 4 | 4 | 4 | 4 | 3.85 | |  | | 80 | 100 | 80 | 64 | 100 | 78.46 | |  | | 8.40 | 11.20 | 11.34 | 3.78 | 9.35 | 8.13 | |
| 16 | AS661564 | Puningxiaomai | 2 | Guangdong |  | 3 | 3 |  | 4 | 3 | 2 | 2 | 2 | 2.58 | |  | | 80 | 40 | 30 | 5 | 62 | 41.57 | |  | | 5.95 | 3.15 | 5.11 | 0.39 | 5.71 | 3.94 | |
| 17 | AS661565 | Wuxuxiaomai | 2 | Guangdong |  | 3 | 4 |  | 4 | 4 | 4 | 4 | 4 | 3.85 | |  | | 100 | 80 | 68 | 50 | 84 | 70.30 | |  | | 10.50 | 11.20 | 9.80 | 2.98 | 5.46 | 7.26 | |
| 18 | AS661566 | Zhangmuxiaomai | 2 | Guangxi |  | 4 | 4 |  | 3 | 4 | 3 | 4 | 4 | 3.50 | |  | | 60 | 80 | 52 | 60 | 100 | 66.73 | |  | | 7.70 | 9.80 | 4.62 | 3.29 | 11.06 | 6.91 | |
| **Additional file 1 *continued*** | | | | | | | | | | | | | | | | | | | | | | | | | | | | | | | | |  |
|  |  |  |  |  |  | Seedling stage | |  | Adult-plant stages | | | | | | | | | | | | | | | | | | | | | | | |  |
| No. | AS_No. ^a^ | Accession Name ^b^ | K=2 | Origin |  | Infection Type (IT) | |  | Infection Type (IT) | | | | | |  | | Disease Severity (DS) | | | | | | |  | | Area under the disease progress curve (AUDPC) | | | | | | |  |
|  |  |  |  |  |  | CYR32 | CYR34 |  | CZ2016 | MY2016 | CZ2017 | MY2017 | CZ2018 | BLUP | |  | | CZ2016 | MY2016 | CZ2017 | MY2017 | CZ2018 | BLUP | |  | | CZ2016 | MY2016 | CZ2017 | MY2017 | CZ2018 | BLUP | |
| 19 | AS661567 | Guipingxiaomai | 2 | Guangxi |  | 3 | 4 |  | 4 | 3 | 3 | 4 | 1 | 2.92 | |  | | 80 | 60 | 80 | 80 | 19 | 57.33 | |  | | 7.00 | 8.40 | 8.82 | 5.04 | 1.75 | 5.59 | |
| 20 | AS661569 | Guangtoumai | 2 | Guangxi |  | 3 | 2 |  | 4 | 3 | 1 | 3 | 4 | 2.97 | |  | | 80 | 40 | 8 | 5 | 72 | 40.24 | |  | | 4.55 | 2.45 | 0.95 | 0.35 | 6.93 | 3.13 | |
| 21 | AS661571 | Sanyuehuang | 2 | Sichuan |  | 4 | 4 |  | 3 | 3 | 3 | 1 | 3 | 2.60 | |  | | 60 | 10 | 11 | 16 | 40 | 27.55 | |  | | 3.85 | 1.40 | 1.30 | 0.77 | 2.35 | 1.99 | |
| 22 | AS661573 | Baimaizi | 1 | Sichuan |  | 3 | 3 |  | 2 | 1 | 3 | 1 | 3 | 2.07 | |  | | 20 | 10 | 15 | 3 | 13 | 14.04 | |  | | 1.23 | 1.23 | 1.58 | 0.11 | 0.74 | 1.11 | |
| 23 | AS661574 | Honghuamaizhong | 1 | Sichuan |  | 4 | 4 |  | 1 | 1 | 1 | 0 | 2 | 1.16 | |  | | 5 | 5 | 5 | 0 | 4 | 6.86 | |  | | 0.35 | 0.70 | 0.70 | 0.00 | 0.21 | 0.59 | |
| 24 | AS661575 | Guangtoumai | 1 | Sichuan |  | 3 | 3 |  | 3 | 3 | 3 | 2 | 3 | 2.77 | |  | | 20 | 20 | 5 | 26 | 37 | 23.37 | |  | | 2.45 | 2.80 | 0.63 | 1.19 | 2.07 | 1.89 | |
| 25 | AS661580 | Erxumai | 1 | Sichuan |  | 3 | 4 |  | 1 | 1 | 1 | 0 | 1 | 0.97 | |  | | 10 | 5 | 3 | 0 | 0 | 6.49 | |  | | 0.88 | 0.70 | 0.63 | 0.00 | 0.00 | 0.62 | |
| 26 | AS661581 | Zhuermai | 1 | Sichuan |  | 3 | 4 |  | 2 | 1 | 1 | 0 | 1 | 1.14 | |  | | 20 | 5 | 19 | 0 | 9 | 12.40 | |  | | 1.23 | 0.70 | 1.82 | 0.04 | 0.49 | 1.00 | |
| 27 | AS661582 | Wuyangmai | 1 | Sichuan |  | 3 | 4 |  | 3 | 3 | 3 | 2 | 3 | 2.77 | |  | | 60 | 40 | 14 | 52 | 49 | 41.67 | |  | | 3.85 | 2.28 | 1.09 | 2.07 | 2.77 | 2.41 | |
| 28 | AS661583 | Huangmaizi | 1 | Sichuan |  | 3 | 4 |  | 1 | 2 | 1 | 0 | 0 | 0.95 | |  | | 5 | 5 | 3 | 0 | 0 | 5.72 | |  | | 0.70 | 0.70 | 0.56 | 0.00 | 0.00 | 0.58 | |
| 29 | AS661584 | Yuzuiweimai | 1 | Sichuan |  | 4 | 3 |  | 1 | 3 | 1 | 0 | 1 | 1.32 | |  | | 10 | 10 | 4 | 0 | 7 | 9.00 | |  | | 0.88 | 1.23 | 0.46 | 0.04 | 0.60 | 0.82 | |
| 30 | AS661585 | Dahuangmai | 1 | Sichuan |  | 3 | 3 |  | 1 | 2 | 2 | 1 | 3 | 1.89 | |  | | 10 | 10 | 6 | 6 | 32 | 15.58 | |  | | 0.88 | 0.88 | 0.74 | 0.39 | 1.58 | 1.07 | |
| 31 | AS661586 | Youtiaomai | 1 | Sichuan |  | 3 | 4 |  | 0 | 1 | 0 | 0 | 2 | 0.81 | |  | | 5 | 5 | 0 | 1 | 11 | 7.72 | |  | | 0.18 | 0.53 | 0.00 | 0.04 | 0.46 | 0.47 | |
| 32 | AS661587 | Dahonghua | 1 | Sichuan |  | 3 | 3 |  | 2 | 2 | 1 | 2 | 2 | 1.87 | |  | | 20 | 10 | 3 | 7 | 20 | 14.35 | |  | | 1.75 | 0.88 | 0.42 | 0.42 | 0.88 | 1.02 | |
| 33 | AS661588 | Nanximai | 1 | Sichuan |  | 3 | 4 |  | 3 | 2 | 1 | 1 | 2 | 1.87 | |  | | 40 | 5 | 2 | 1 | 22 | 15.74 | |  | | 1.93 | 1.23 | 0.42 | 0.07 | 1.54 | 1.19 | |
| 34 | AS661589 | Guangtoumai | 1 | Sichuan |  | 3 | 4 |  | 2 | - | 1 | 1 | 1 | 1.44 | |  | | 20 | - | 2 | 3 | 2 | 10.73 | |  | | 0.70 | - | 0.53 | 0.25 | 0.21 | 0.76 | |
| 35 | AS661590 | Dongmaier | 1 | Sichuan |  | 3 | 3 |  | 1 | 2 | 0 | 0 | 2 | 1.16 | |  | | 5 | 5 | 1 | 0 | 22 | 9.99 | |  | | 0.70 | 0.70 | 0.42 | 0.00 | 1.37 | 0.85 | |
| 36 | AS661592 | Zaohonghua | 1 | Sichuan |  | 3 | 3 |  | 2 | 3 | 2 | 3 | 2 | 2.40 | |  | | 20 | 20 | 7 | 44 | 18 | 22.94 | |  | | 1.75 | 1.75 | 0.77 | 1.89 | 1.02 | 1.51 | |
| 37 | AS661593 | Zaohuangmai | 1 | Sichuan |  | 3 | 3 |  | 3 | 3 | 2 | 2 | 3 | 2.60 | |  | | 60 | 20 | 8 | 5 | 28 | 24.41 | |  | | 5.60 | 1.75 | 0.81 | 0.25 | 1.72 | 2.04 | |
| **Additional file 1 *continued*** | | | | | | | | | | | | | | | | | | | | | | | | | | | | | | | | |  |
|  |  |  |  |  |  | Seedling stage | |  | Adult-plant stages | | | | | | | | | | | | | | | | | | | | | | | |  |
| No. | AS_No. ^a^ | Accession Name ^b^ | K=2 | Origin |  | Infection Type (IT) | |  | Infection Type (IT) | | | | | |  | | Disease Severity (DS) | | | | | | |  | | Area under the disease progress curve (AUDPC) | | | | | | |  |
|  |  |  |  |  |  | CYR32 | CYR34 |  | CZ2016 | MY2016 | CZ2017 | MY2017 | CZ2018 | BLUP | |  | | CZ2016 | MY2016 | CZ2017 | MY2017 | CZ2018 | BLUP | |  | | CZ2016 | MY2016 | CZ2017 | MY2017 | CZ2018 | BLUP | |
| 38 | AS661594 | Yizaixiaomai | 1 | Sichuan |  | 3 | 3 |  | 0 | 1 | 0 | 0 | 1 | 0.61 | |  | | 0 | 5 | 0 | 0 | 0 | 4.48 | |  | | 0.00 | 0.53 | 0.00 | 0.00 | 0.00 | 0.34 | |
| 39 | AS661596 | Hongtiaomai | 1 | Sichuan |  | 4 | 3 |  | 4 | 4 | 3 | 3 | 3 | 3.31 | |  | | 60 | 80 | 12 | 80 | 40 | 51.62 | |  | | 5.25 | 9.80 | 2.45 | 4.55 | 2.56 | 4.54 | |
| 40 | AS661597 | Guangtou | 2 | Sichuan |  | 3 | 3 |  | 4 | 3 | 4 | 4 | 4 | 3.68 | |  | | 100 | 60 | 100 | 56 | 92 | 74.43 | |  | | 8.40 | 5.25 | 11.90 | 3.36 | 7.49 | 6.75 | |
| 41 | AS661598 | Guangtoumai | 1 | Sichuan |  | 3 | 4 |  | 3 | 3 | 1 | 1 | 1 | 1.85 | |  | | 40 | 10 | 4 | 4 | 1 | 13.11 | |  | | 2.45 | 1.40 | 0.67 | 0.39 | 0.11 | 1.11 | |
| 42 | AS661599 | Yupi | 2 | Sichuan |  | 3 | 4 |  | 4 | 4 | 3 | 4 | 4 | 3.68 | |  | | 80 | 100 | 52 | 80 | 100 | 76.97 | |  | | 10.50 | 13.30 | 8.68 | 4.27 | 12.46 | 9.13 | |
| 43 | AS661600 | Baimai | 2 | Sichuan |  | 4 | 3 |  | 4 | 3 | 3 | 2 | 3 | 2.95 | |  | | 80 | 40 | 24 | 34 | 64 | 46.23 | |  | | 7.70 | 3.50 | 2.21 | 1.61 | 4.31 | 3.71 | |
| 44 | AS661602 | Hongxumai | 2 | Sichuan |  | 3 | 3 |  | 1 | 1 | 0 | 1 | 1 | 0.97 | |  | | 5 | 5 | 0 | 1 | 3 | 6.06 | |  | | 0.18 | 0.70 | 0.39 | 0.07 | 0.18 | 0.51 | |
| 45 | AS661604 | Doumai | 1 | Sichuan |  | 3 | 4 |  | 2 | 1 | 1 | 0 | 1 | 1.14 | |  | | 10 | 5 | 4 | 0 | 0 | 6.65 | |  | | 1.40 | 0.88 | 0.67 | 0.00 | 0.07 | 0.76 | |
| 46 | AS661605 | Lushanmai | 1 | Sichuan |  | 1 | 1 |  | 1 | 1 | 0 | 0 | 1 | 0.79 | |  | | 5 | 5 | 0 | 0 | 0 | 5.25 | |  | | 0.53 | 0.53 | 0.00 | 0.00 | 0.00 | 0.43 | |
| 47 | AS661606 | Lanmai | 1 | Sichuan |  | 3 | 3 |  | 1 | 1 | 0 | 0 | 2 | 0.98 | |  | | 5 | 5 | 0 | 0 | 8 | 6.92 | |  | | 0.53 | 0.70 | 0.28 | 0.00 | 1.12 | 0.74 | |
| 48 | AS661609 | Tuotuomai | 1 | Sichuan |  | 3 | 3 |  | 1 | 3 | 0 | 0 | 1 | 1.14 | |  | | 5 | 5 | 0 | 0 | 6 | 6.50 | |  | | 0.70 | 0.53 | 0.32 | 0.00 | 0.56 | 0.63 | |
| 49 | AS661610 | Honghuaguangtou | 1 | Sichuan |  | 3 | 1 |  | 4 | 3 | 2 | 0 | 3 | 2.42 | |  | | 60 | 60 | 11 | 0 | 21 | 29.67 | |  | | 4.20 | 4.20 | 1.16 | 0.00 | 1.09 | 2.10 | |
| 50 | AS661611 | Wulangxiexiaomai | 1 | Sichuan |  | 3 | 4 |  | 1 | 2 | 0 | 1 | 1 | 1.14 | |  | | 5 | 5 | 1 | 2 | 9 | 7.64 | |  | | 0.70 | 0.70 | 0.49 | 0.07 | 1.09 | 0.81 | |
| 51 | AS661612 | Changxuxuqiaomai | 1 | Sichuan |  | 3 | 3 |  | 3 | 1 | 1 | 1 | 1 | 1.50 | |  | | 40 | 5 | 2 | 7 | 7 | 13.70 | |  | | 2.10 | 0.70 | 0.53 | 0.39 | 1.05 | 1.10 | |
| 52 | AS661613 | Qingquanxiaomai | 2 | Sichuan |  | 3 | 3 |  | 4 | 1 | 4 | 4 | 2 | 2.93 | |  | | 80 | 5 | 64 | 80 | 16 | 44.41 | |  | | 7.35 | 0.70 | 6.51 | 4.62 | 1.09 | 3.74 | |
| 53 | AS661615 | Yuweimai | 1 | Sichuan |  | 4 | 4 |  | 1 | 2 | 1 | 1 | 2 | 1.52 | |  | | 5 | 5 | 4 | 5 | 5 | 7.81 | |  | | 0.70 | 0.70 | 0.67 | 0.35 | 0.53 | 0.77 | |
| 54 | AS661616 | Hechuanmai | 1 | Sichuan |  | 4 | 4 |  | 1 | 2 | 1 | 1 | 1 | 1.32 | |  | | 10 | 10 | 5 | 1 | 0 | 7.88 | |  | | 0.88 | 0.88 | 0.70 | 0.04 | 0.00 | 0.67 | |
| 55 | AS661617 | Huayangxiaomai | 1 | Sichuan |  | 4 | 4 |  | 0 | 0 | 0 | 0 | 1 | 0.44 | |  | | 5 | 0 | 0 | 0 | 0 | 4.36 | |  | | 0.18 | 0.00 | 0.14 | 0.00 | 0.00 | 0.30 | |
| 56 | AS661618 | Wumangmai | 1 | Sichuan |  | 3 | 3 |  | 3 | 3 | 3 | 1 | 3 | 2.60 | |  | | 20 | 40 | 14 | 9 | 36 | 25.09 | |  | | 2.28 | 4.90 | 1.16 | 0.49 | 3.71 | 2.53 | |
| **Additional file 1 *continued*** | | | | | | | | | | | | | | | | | | | | | | | | | | | | | | | | |  |
|  |  |  |  |  |  | Seedling stage | |  | Adult-plant stages | | | | | | | | | | | | | | | | | | | | | | | |  |
| No. | AS_No. ^a^ | Accession Name ^b^ | K=2 | Origin |  | Infection Type (IT) | |  | Infection Type (IT) | | | | | |  | | Disease Severity (DS) | | | | | | |  | | Area under the disease progress curve (AUDPC) | | | | | | |  |
|  |  |  |  |  |  | CYR32 | CYR34 |  | CZ2016 | MY2016 | CZ2017 | MY2017 | CZ2018 | BLUP | |  | | CZ2016 | MY2016 | CZ2017 | MY2017 | CZ2018 | BLUP | |  | | CZ2016 | MY2016 | CZ2017 | MY2017 | CZ2018 | BLUP | |
| 57 | AS661619 | Huanghuaxiaomai | 2 | Sichuan |  | 4 | 3 |  | 3 | 3 | 4 | 3 | 2 | 2.93 | |  | | 80 | 60 | 68 | 48 | 56 | 57.45 | |  | | 3.33 | 5.60 | 5.81 | 1.93 | 3.57 | 3.84 | |
| 58 | AS661620 | Guangguangtou | 1 | Sichuan |  | 4 | 3 |  | 2 | 2 | 3 | 2 | 2 | 2.22 | |  | | 40 | 10 | 19 | 9 | 6 | 17.37 | |  | | 2.28 | 0.88 | 1.86 | 0.46 | 0.77 | 1.34 | |
| 59 | AS661621 | Danaoke | 1 | Sichuan |  | 3 | 4 |  | 3 | 3 | 1 | 1 | 4 | 2.44 | |  | | 60 | 40 | 5 | 3 | 58 | 33.40 | |  | | 2.45 | 1.93 | 0.39 | 0.11 | 4.59 | 2.05 | |
| 60 | AS661622 | Yangmai | 1 | Sichuan |  | 4 | 4 |  | 2 | 2 | 2 | 0 | 1 | 1.50 | |  | | 10 | 5 | 6 | 0 | 4 | 7.79 | |  | | 0.88 | 0.70 | 0.81 | 0.04 | 0.35 | 0.74 | |
| 61 | AS661625 | Hongkejiang | 1 | Sichuan |  | 3 | 3 |  | 2 | 3 | 1 | 2 | 4 | 2.44 | |  | | 20 | 60 | 5 | 7 | 44 | 28.57 | |  | | 1.40 | 6.65 | 0.88 | 0.46 | 2.21 | 2.31 | |
| 62 | AS661626 | Baikejiang | 2 | Sichuan |  | 3 | 4 |  | 3 | 2 | 2 | 2 | 4 | 2.61 | |  | | 40 | 10 | 5 | 5 | 44 | 22.39 | |  | | 2.98 | 1.40 | 0.70 | 0.35 | 4.63 | 2.15 | |
| 63 | AS661627 | Bazhouxiaomai | 1 | Sichuan |  | 4 | 4 |  | 1 | 2 | 1 | 1 | 2 | 1.52 | |  | | 10 | 5 | 6 | 5 | 5 | 8.89 | |  | | 0.70 | 0.70 | 0.74 | 0.21 | 0.67 | 0.79 | |
| 64 | AS661629 | Honghuamai | 1 | Sichuan |  | 3 | 4 |  | 3 | 3 | 2 | 1 | 3 | 2.42 | |  | | 40 | 10 | 5 | 4 | 36 | 20.55 | |  | | 2.28 | 1.23 | 0.91 | 0.21 | 1.89 | 1.44 | |
| 65 | AS661630 | Chikezhuomai | 2 | Sichuan |  | 3 | 3 |  | 3 | 3 | 1 | 1 | 3 | 2.24 | |  | | 60 | 5 | 5 | 1 | 48 | 24.72 | |  | | 3.68 | 1.58 | 0.70 | 0.21 | 3.61 | 2.06 | |
| 66 | AS661631 | Beibeixiaomai | 1 | Sichuan |  | 3 | 3 |  | 1 | 1 | 3 | 1 | 2 | 1.69 | |  | | 5 | 5 | 5 | 1 | 5 | 7.25 | |  | | 0.70 | 0.70 | 0.49 | 0.07 | 0.32 | 0.65 | |
| 67 | AS661633 | Huoshaomai | 1 | Sichuan |  | 4 | 4 |  | 2 | 2 | 3 | 1 | 2 | 2.05 | |  | | 40 | 10 | 13 | 3 | 26 | 19.53 | |  | | 2.45 | 1.40 | 1.68 | 0.18 | 2.00 | 1.64 | |
| 68 | AS661636 | Dongmai | 1 | Sichuan |  | 3 | 4 |  | 2 | 1 | 2 | 1 | 1 | 1.50 | |  | | 20 | 5 | 4 | 3 | 13 | 11.44 | |  | | 1.58 | 0.70 | 1.19 | 0.32 | 1.12 | 1.13 | |
| 69 | AS661637 | Zaodongmai | 1 | Sichuan |  | 3 | 4 |  | 2 | 1 | 0 | 1 | 1 | 1.14 | |  | | 20 | 5 | 0 | 4 | 3 | 8.92 | |  | | 1.58 | 0.70 | 0.53 | 0.39 | 0.11 | 0.82 | |
| 70 | AS661639 | Hongkekemaizi | 1 | Sichuan |  | 3 | 4 |  | 3 | 3 | 3 | 1 | 1 | 2.21 | |  | | 20 | 20 | 12 | 10 | 5 | 14.94 | |  | | 1.58 | 1.23 | 1.82 | 0.49 | 0.32 | 1.19 | |
| 71 | AS661640 | Heshangmai | 2 | Sichuan |  | 3 | 3 |  | 1 | 2 | 1 | 0 | 1 | 1.14 | |  | | 10 | 10 | 3 | 0 | 7 | 8.84 | |  | | 0.70 | 0.88 | - | 0.00 | 0.25 | 0.68 | |
| 72 | AS661641 | Hongpimai | 1 | Sichuan |  | 3 | 3 |  | 0 | 1 | 1 | 1 | 2 | 1.16 | |  | | 5 | 5 | 5 | 4 | 3 | 7.37 | |  | | 0.70 | 0.70 | 0.63 | 0.28 | 0.46 | 0.74 | |
| 73 | AS661642 | Yicuomao | 2 | Sichuan |  | 3 | 3 |  | 2 | 3 | 3 | 2 | 3 | 2.60 | |  | | 20 | 40 | 13 | 12 | 22 | 22.56 | |  | | 1.40 | 2.98 | 0.98 | 0.60 | 1.65 | 1.61 | |
| 74 | AS661643 | Baixiaomai | 1 | Sichuan |  | 3 | 4 |  | 2 | 2 | 1 | 1 | 1 | 1.50 | |  | | 20 | 10 | 5 | 2 | 7 | 11.06 | |  | | 1.23 | 0.88 | 0.56 | 0.18 | 0.67 | 0.87 | |
| 75 | AS661644 | Fangmai | 1 | Sichuan |  | 4 | 4 |  | 2 | 3 | 3 | 2 | 3 | 2.60 | |  | | 20 | 40 | 6 | 21 | 36 | 25.99 | |  | | 1.75 | 3.15 | 0.74 | 1.26 | 2.31 | 1.91 | |
| **Additional file 1 *continued*** | | | | | | | | | | | | | | | | | | | | | | | | | | | | | | | | |  |
|  |  |  |  |  |  | Seedling stage | |  | Adult-plant stages | | | | | | | | | | | | | | | | | | | | | | | |  |
| No. | AS_No. ^a^ | Accession Name ^b^ | K=2 | Origin |  | Infection Type (IT) | |  | Infection Type (IT) | | | | | |  | | Disease Severity (DS) | | | | | | |  | | Area under the disease progress curve (AUDPC) | | | | | | |  |
|  |  |  |  |  |  | CYR32 | CYR34 |  | CZ2016 | MY2016 | CZ2017 | MY2017 | CZ2018 | BLUP | |  | | CZ2016 | MY2016 | CZ2017 | MY2017 | CZ2018 | BLUP | |  | | CZ2016 | MY2016 | CZ2017 | MY2017 | CZ2018 | BLUP | |
| 76 | AS661645 | Baidongmai | 1 | Sichuan |  | 3 | 4 |  | 2 | 1 | 1 | 0 | 3 | 1.53 | |  | | 20 | 5 | 4 | 0 | 23 | 12.98 | |  | | 1.40 | 0.70 | 0.74 | 0.00 | 1.61 | 1.07 | |
| 77 | AS661647 | Laolaihong | 1 | Sichuan |  | 3 | 4 |  | 3 | 3 | 1 | 1 | 3 | 2.24 | |  | | 40 | 20 | 2 | 3 | 24 | 19.19 | |  | | 3.15 | 2.45 | 1.05 | 0.18 | 1.68 | 1.76 | |
| 78 | AS661648 | Baihuamai | 2 | Sichuan |  | 3 | 3 |  | 1 | 2 | 1 | 1 | 3 | 1.71 | |  | | 20 | 10 | 14 | 8 | 56 | 23.66 | |  | | 1.58 | 0.88 | 1.00 | 0.67 | 5.08 | 2.03 | |
| 79 | AS661652 | Sanyuehuang | 1 | Sichuan |  | 3 | 4 |  | 1 | 2 | 3 | 1 | 2 | 1.87 | |  | | 10 | 5 | 6 | 3 | 6 | 8.74 | |  | | 0.88 | 0.70 | 0.74 | 0.14 | 0.39 | 0.75 | |
| 80 | AS661654 | Honghuaxuxumai | 2 | Sichuan |  | 3 | 3 |  | 2 | 1 | 1 | 1 | 3 | 1.71 | |  | | 10 | 5 | 2 | 1 | 22 | 11.09 | |  | | 1.23 | 0.70 | 0.60 | 0.11 | 1.82 | 1.08 | |
| 81 | AS661655 | Baimaier | 2 | Sichuan |  | 3 | 3 |  | 1 | 2 | 3 | 1 | 4 | 2.26 | |  | | 5 | 10 | 17 | 3 | 76 | 25.13 | |  | | 0.70 | 0.88 | 1.12 | 0.32 | 3.61 | 1.53 | |
| 82 | AS661656 | Hongkebaidongmai | 1 | Sichuan |  | 4 | 3 |  | 1 | 2 | 1 | 0 | 2 | 1.34 | |  | | 5 | 5 | 4 | 0 | 10 | 7.95 | |  | | 1.93 | 0.70 | 0.32 | 0.04 | 0.35 | 0.83 | |
| 83 | AS661657 | Yuqiumai | 2 | Sichuan |  | 2 | 1 |  | 1 | 3 | 1 | 1 | 1 | 1.50 | |  | | 10 | 20 | 3 | 1 | 7 | 10.81 | |  | | 0.70 | 1.58 | 0.42 | 0.04 | 0.60 | 0.84 | |
| 84 | AS661658 | Baikexuxusanyuehuang | 1 | Sichuan |  | 3 | 3 |  | 2 | 3 | 1 | 1 | 2 | 1.87 | |  | | 20 | 10 | 5 | 9 | 8 | 12.52 | |  | | 1.58 | 0.88 | 0.63 | 0.49 | 0.56 | 0.98 | |
| 85 | AS661661 | Paidengmai | 2 | Sichuan |  | 4 | 4 |  | 3 | 1 | 0 | 1 | 3 | 1.71 | |  | | 40 | 5 | 0 | 2 | 28 | 16.86 | |  | | 3.50 | 0.70 | 0.32 | 0.07 | 3.01 | 1.67 | |
| 86 | AS661662 | Yumai | 2 | Guizhou |  | 3 | 3 |  | 3 | 4 | 4 | 4 | 4 | 3.68 | |  | | 60 | 100 | 100 | 84 | 100 | 82.03 | |  | | 7.70 | 13.30 | 12.60 | 5.74 | 7.25 | 8.47 | |
| 87 | AS661663 | Gaoganhongmai | 2 | Guizhou |  | 3 | 3 |  | 3 | 4 | 4 | 4 | 3 | 3.48 | |  | | 40 | 80 | 80 | 100 | 30 | 60.55 | |  | | 3.15 | 7.70 | 5.74 | 5.18 | 1.82 | 4.33 | |
| 88 | AS661664 | Dahongmai | 2 | Guizhou |  | 4 | 3 |  | 3 | 4 | 3 | 2 | 3 | 2.95 | |  | | 40 | 80 | 11 | 11 | 36 | 35.22 | |  | | 3.50 | 4.90 | 1.75 | 0.60 | 1.47 | 2.39 | |
| 89 | AS661665 | Yuqiumai | 2 | Guizhou |  | 4 | 3 |  | 4 | 4 | 2 | 1 | 3 | 2.77 | |  | | 60 | 80 | 6 | 4 | 20 | 32.97 | |  | | 4.20 | 8.40 | 1.19 | 0.32 | 0.98 | 2.85 | |
| 90 | AS661666 | Xinianmai | 2 | Guizhou |  | 3 | 3 |  | 1 | 2 | 0 | 0 | 1 | 0.97 | |  | | 5 | 10 | 0 | 0 | 1 | 6.35 | |  | | 0.70 | 0.88 | 0.25 | 0.00 | 0.07 | 0.57 | |
| 91 | AS661667 | Caoxiepian | 2 | Guizhou |  | 3 | 3 |  | 3 | 3 | 1 | 1 | 3 | 2.24 | |  | | 20 | 40 | 5 | 3 | 24 | 20.13 | |  | | 1.58 | 1.93 | 0.70 | 0.25 | 1.72 | 1.37 | |
| 92 | AS661668 | Huimai | 2 | Guizhou |  | 3 | 3 |  | 4 | 3 | 3 | 3 | 4 | 3.32 | |  | | 80 | 60 | 18 | 80 | 60 | 56.24 | |  | | 9.10 | 5.60 | 1.54 | 3.57 | 4.13 | 4.49 | |
| 93 | AS661669 | Youmai | 2 | Guizhou |  | 4 | 4 |  | 4 | 4 | 4 | 4 | 4 | 3.85 | |  | | 100 | 100 | 100 | 80 | 100 | 87.51 | |  | | 11.20 | 12.60 | 10.64 | 6.02 | 11.06 | 9.47 | |
| 94 | AS661670 | Huanghuamai | 1 | Guizhou |  | 4 | 4 |  | 3 | 3 | 3 | 1 | 3 | 2.60 | |  | | 40 | 40 | 29 | 8 | 64 | 36.16 | |  | | 4.55 | 2.45 | 5.50 | 0.53 | 3.78 | 3.26 | |
| **Additional file 1 *continued*** | | | | | | | | | | | | | | | | | | | | | | | | | | | | | | | | |  |
|  |  |  |  |  |  | Seedling stage | |  | Adult-plant stages | | | | | | | | | | | | | | | | | | | | | | | |  |
| No. | AS_No. ^a^ | Accession Name ^b^ | K=2 | Origin |  | Infection Type (IT) | |  | Infection Type (IT) | | | | | |  | | Disease Severity (DS) | | | | | | |  | | Area under the disease progress curve (AUDPC) | | | | | | |  |
|  |  |  |  |  |  | CYR32 | CYR34 |  | CZ2016 | MY2016 | CZ2017 | MY2017 | CZ2018 | BLUP | |  | | CZ2016 | MY2016 | CZ2017 | MY2017 | CZ2018 | BLUP | |  | | CZ2016 | MY2016 | CZ2017 | MY2017 | CZ2018 | BLUP | |
| 95 | AS661671 | Guangtoumai | 2 | Guizhou |  | 2 | 2 |  | 2 | 2 | 1 | 0 | 1 | 1.32 | |  | | 10 | 5 | 1 | 0 | 3 | 6.81 | |  | | 0.70 | 0.70 | 0.04 | 0.00 | 0.11 | 0.52 | |
| 96 | AS661675 | Hongmangmai | 2 | Guizhou |  | 2 | 3 |  | 1 | 1 | 0 | 1 | 3 | 1.36 | |  | | 5 | 5 | 2 | 1 | 19 | 9.70 | |  | | 0.70 | 0.70 | 0.39 | 0.04 | 1.89 | 0.96 | |
| 97 | AS661676 | Liulengmai | 2 | Guizhou |  | 3 | 2 |  | 1 | 1 | 1 | 0 | 2 | 1.16 | |  | | 20 | 5 | 5 | 0 | 36 | 15.84 | |  | | 0.70 | 0.70 | 0.63 | 0.07 | 2.28 | 1.09 | |
| 98 | AS661678 | Baihuamai | 2 | Guizhou |  | 3 | 3 |  | 1 | 3 | 0 | 0 | 3 | 1.53 | |  | | 20 | 20 | 1 | 0 | 19 | 14.36 | |  | | 1.05 | 2.28 | 0.28 | 0.00 | 1.02 | 1.08 | |
| 99 | AS661679 | Tiekemai | 2 | Yunnan |  | 4 | 3 |  | 0 | 1 | 0 | 0 | 0 | 0.42 | |  | | 5 | 0 | 0 | 0 | 0 | 4.36 | |  | | 0.18 | 0.00 | 0.00 | 0.00 | 0.00 | 0.28 | |
| 100 | AS661681 | Fengqingxiaomai | 2 | Yunnan |  | 4 | 3 |  | 2 | 1 | 1 | 1 | 3 | 1.71 | |  | | 10 | 5 | 5 | 3 | 24 | 12.33 | |  | | 0.88 | 0.70 | 0.53 | 0.28 | 0.84 | 0.83 | |
| 101 | AS661682 | Zhushimai | 1 | Yunnan |  | 4 | 2 |  | 3 | 3 | 1 | 2 | 3 | 2.42 | |  | | 20 | 80 | 12 | 15 | 24 | 30.49 | |  | | 1.23 | 4.38 | 0.81 | 0.77 | 0.98 | 1.68 | |
| 102 | AS661683 | Zimai | 1 | Yunnan |  | 4 | 4 |  | 0 | 0 | 0 | 0 | 0 | 0.24 | |  | | 5 | 0 | 0 | 0 | 0 | 4.36 | |  | | 0.18 | 0.00 | 0.14 | 0.00 | 0.00 | 0.30 | |
| 103 | AS661684 | Yangmaizi | 2 | Yunnan |  | 3 | 3 |  | 2 | 3 | 3 | 1 | 3 | 2.42 | |  | | 10 | 60 | 37 | 11 | 38 | 31.45 | |  | | 0.88 | 3.68 | 1.89 | 0.53 | 1.54 | 1.76 | |
| 104 | AS661712 | Shangxuezao | 2 | Fujian |  | 3 | 4 |  | 4 | 4 | 4 | 4 | 4 | 3.85 | |  | | 100 | 100 | 76 | 100 | 92 | 85.70 | |  | | 11.90 | 11.20 | 9.52 | 5.32 | 8.30 | 8.45 | |
| 105 | AS661713 | Guwanchisui | 2 | Fujian |  | 3 | 3 |  | 1 | 2 | 2 | 3 | 4 | 2.44 | |  | | 5 | 5 | 13 | 4 | 56 | 19.63 | |  | | 0.53 | 0.70 | 0.91 | 0.25 | 2.91 | 1.27 | |
| 106 | AS661715 | Heshangmai | 2 | Fujian |  | 3 | 3 |  | 4 | 4 | 3 | 4 | 4 | 3.68 | |  | | 80 | 80 | 68 | 6 | 68 | 56.02 | |  | | 10.50 | 8.40 | 8.82 | 0.35 | 4.41 | 5.95 | |
| 107 | AS661716 | Guangchangliuyemai | 2 | Jiangxi |  | 3 | 3 |  | 3 | 3 | 3 | 1 | 4 | 2.79 | |  | | 40 | 40 | 32 | 2 | 22 | 26.82 | |  | | 3.15 | 2.45 | 2.84 | 0.14 | 1.86 | 2.10 | |
| 108 | AS661763 | Guangtoumai | 2 | Guangdong |  | 3 | 4 |  | 4 | 4 | 3 | 4 | 3 | 3.48 | |  | | 100 | 80 | 56 | 9 | 72 | 58.63 | |  | | 9.80 | 9.80 | 6.86 | 0.49 | 3.29 | 5.52 | |
| 109 | AS661764 | Bendixiaomai | 2 | Guangxi |  | 3 | 4 |  | 4 | 3 | 2 | 4 | 3 | 3.13 | |  | | 80 | 20 | 26 | 11 | 40 | 33.87 | |  | | 5.95 | 2.45 | 3.92 | 0.81 | 2.84 | 3.08 | |
| 110 | AS661771 | Tuoermai | 1 | Sichuan |  | 4 | 3 |  | 0 | 1 | 0 | 0 | 1 | 0.61 | |  | | 5 | 5 | 1 | 0 | 10 | 7.49 | |  | | 0.70 | 0.70 | 0.28 | 0.00 | 0.81 | 0.71 | |
| 111 | AS661773 | Guangtouxiaomai | 1 | Sichuan |  | 3 | 3 |  | 3 | 3 | 3 | 1 | 3 | 2.60 | |  | | 40 | 10 | 30 | 2 | 52 | 27.40 | |  | | 1.75 | 0.88 | 2.66 | 0.11 | 3.29 | 1.86 | |
| 112 | AS661774 | Guangtouxiaomai | 2 | Sichuan |  | 4 | 3 |  | 3 | 3 | 2 | 1 |  | 2.35 | |  | | 40 | 60 | 8 | 5 | 88 | 40.93 | |  | | 1.75 | 3.68 | 0.63 | 0.28 | 5.81 | 2.56 | |
| 113 | AS661777 | Zhenixiaomai | 2 | Sichuan |  | 1 | 2 |  | 3 | 2 | 3 | 0 | 1 | 1.85 | |  | | - | 20 | 15 | 0 | 6 | 14.21 | |  | | - | 1.05 | 0.77 | 0.00 | 0.42 | 0.90 | |
| **Additional file 1 *continued*** | | | | | | | | | | | | | | | | | | | | | | | | | | | | | | | | |  |
|  |  |  |  |  |  | Seedling stage | |  | Adult-plant stages | | | | | | | | | | | | | | | | | | | | | | | |  |
| No. | AS_No. ^a^ | Accession Name ^b^ | K=2 | Origin |  | Infection Type (IT) | |  | Infection Type (IT) | | | | | |  | | Disease Severity (DS) | | | | | | |  | | Area under the disease progress curve (AUDPC) | | | | | | |  |
|  |  |  |  |  |  | CYR32 | CYR34 |  | CZ2016 | MY2016 | CZ2017 | MY2017 | CZ2018 | BLUP | |  | | CZ2016 | MY2016 | CZ2017 | MY2017 | CZ2018 | BLUP | |  | | CZ2016 | MY2016 | CZ2017 | MY2017 | CZ2018 | BLUP | |
| 114 | AS661779 | Qianqianmai | 2 | Guizhou |  | 3 | 3 |  | 1 | 1 | 0 | 0 | 1 | 0.79 | |  | | 5 | 5 | 1 | 0 | 4 | 6.24 | |  | | 0.53 | 0.70 | 0.53 | 0.00 | 0.53 | 0.66 | |
| 115 | AS661780 | Wuhuaxiaomai | 2 | Guizhou |  | 3 | 2 |  | 4 | 4 | 4 | 4 | 4 | 3.85 | |  | | 80 | 100 | 76 | 75 | 64 | 72.31 | |  | | 5.95 | 11.20 | 6.93 | 4.29 | 5.22 | 6.17 | |
| 116 | AS661781 | Daheimai | 2 | Yunnan |  | 4 | 4 |  | 2 | 3 | 4 | 1 | 2 | 2.40 | |  | | 20 | 60 | 22 | 11 | 17 | 26.30 | |  | | 1.58 | 5.60 | 1.93 | 0.56 | 0.70 | 2.04 | |
| 117 | AS661782 | Guangyiwuyuemai-8 | 2 | Yunnan |  | 4 | 4 |  | 0 | 1 | 0 | 0 | 1 | 0.61 | |  | | - | 5 | 0 | 0 | 0 | 6.28 | |  | | - | 0.18 | 0.25 | 0.00 | 0.00 | 0.49 | |
| 118 | AS661783 | Wumulongxiaobaimai | 2 | Yunnan |  | 3 | 3 |  | 1 | 3 | 1 | 1 | 1 | 1.50 | |  | | 5 | 10 | 5 | 6 | 18 | 11.74 | |  | | 0.53 | 0.88 | 0.70 | 0.39 | 0.70 | 0.82 | |
| 119 | AS661784 | Bendixiaomai | 1 | Yunnan |  | 4 | 3 |  | 3 | 3 | 2 | 2 | 3 | 2.60 | |  | | 40 | 40 | 12 | 24 | 48 | 33.05 | |  | | 2.45 | 3.15 | 1.61 | 1.23 | 2.31 | 2.17 | |
| 120 | AS661785 | Bendiyoumangxiaomai | 1 | Yunnan |  | 0 | 3 |  | 0 | 0 | 0 | 0 | 2 | 0.63 | |  | | 0 | 0 | 0 | 0 | 11 | 5.88 | |  | | 0.00 | 0.00 | 0.00 | 0.00 | 0.39 | 0.33 | |
| 121 | AS661786 | Bendizimai | 1 | Yunnan |  | 3 | 4 |  | 3 | 3 | 3 | 1 | 3 | 2.60 | |  | | 60 | 40 | 44 | 6 | 56 | 39.57 | |  | | 3.68 | 5.60 | 2.73 | 0.35 | 3.12 | 3.01 | |
| 122 | AS661790 | Youmanghuakemai | 2 | Yunnan |  | 3 | 4 |  | 3 | 3 | 4 | 3 | 1 | 2.74 | |  | | 60 | 40 | 16 | 29 | 7 | 29.14 | |  | | 2.98 | 2.28 | 1.05 | 1.51 | 0.25 | 1.63 | |
| 123 | AS661791 | Guangtoubaikemai | 2 | Yunnan |  | 3 | 3 |  | 2 | 1 | 4 | 0 | 1 | 1.68 | |  | | - | 5 | 17 | 0 | 0 | 9.47 | |  | | - | 0.53 | 0.60 | 0.00 | 0.21 | 0.69 | |
| 124 | AS661792 | Guangtoumai | 2 | Yunnan |  | 4 | 4 |  | 0 | 1 | 0 | 0 | 1 | 0.61 | |  | | 5 | 5 | 0 | 0 | 2 | 5.67 | |  | | 0.18 | 0.18 | 0.07 | 0.00 | 0.11 | 0.34 | |
| 125 | AS661793 | Guangtoumai-2 | 2 | Yunnan |  | 3 | 3 |  | 3 | 3 | 3 | 4 | 4 | 3.32 | |  | | 60 | 40 | 45 | 80 | 88 | 59.58 | |  | | 5.25 | 2.98 | 4.24 | 4.34 | 5.64 | 4.30 | |
| 126 | AS661794 | Hongganguangtoumai | 1 | Yunnan |  | 4 | 3 |  | 4 | 3 | 3 | 1 | 3 | 2.77 | |  | | 80 | 40 | 8 | 4 | 30 | 31.32 | |  | | 5.95 | 4.90 | 1.16 | 0.25 | 2.42 | 2.84 | |
| 127 | AS661795 | Huakemai | 2 | Yunnan |  | 3 | 3 |  | 0 | 1 | 1 | 0 | 1 | 0.79 | |  | | 5 | 5 | 3 | 0 | 0 | 5.72 | |  | | 0.18 | 0.53 | 0.32 | 0.00 | 0.00 | 0.42 | |
| 128 | AS661796 | Ruankemai | 2 | Yunnan |  | 3 | 3 |  | 0 | 2 | 0 | 1 | 1 | 0.97 | |  | | 5 | 5 | 0 | 1 | 2 | 5.85 | |  | | 0.70 | 0.70 | 0.53 | 0.04 | 0.11 | 0.61 | |
| 129 | AS661798 | Chunmai | 2 | Yunnan |  | 3 | 3 |  | 4 | 3 | 2 | 2 | 4 | 2.97 | |  | | 100 | 60 | 11 | 14 | 100 | 54.80 | |  | | 7.00 | 5.08 | 1.37 | 0.67 | 11.90 | 5.17 | |
| 130 | AS661822 | Baiyanglazi | 1 | Shaanxi |  | 3 | 3 |  | 1 | 1 | 0 | 0 | 2 | 0.98 | |  | | 10 | 5 | 0 | 0 | 21 | 10.40 | |  | | 0.35 | 0.53 | 0.00 | 0.00 | 1.16 | 0.64 | |
| 131 | AS661839 | Honghuamai | 1 | Shaanxi |  | 3 | 4 |  | 0 | 0 | 0 | 0 | 2 | 0.63 | |  | | 0 | 0 | 0 | 0 | 14 | 6.50 | |  | | 0.00 | 0.00 | 0.00 | 0.00 | 0.84 | 0.43 | |
| 132 | AS661842 | Hongxingliu | 1 | Shaanxi |  | 3 | 4 |  | 1 | 0 | 0 | 0 | 3 | 1.00 | |  | | 10 | 0 | 1 | 0 | 26 | 10.70 | |  | | 0.35 | 0.18 | 0.21 | 0.00 | 1.26 | 0.64 | |
| **Additional file 1 *continued*** | | | | | | | | | | | | | | | | | | | | | | | | | | | | | | | | |  |
|  |  |  |  |  |  | Seedling stage | |  | Adult-plant stages | | | | | | | | | | | | | | | | | | | | | | | |  |
| No. | AS_No. ^a^ | Accession Name ^b^ | K=2 | Origin |  | Infection Type (IT) | |  | Infection Type (IT) | | | | | |  | | Disease Severity (DS) | | | | | | |  | | Area under the disease progress curve (AUDPC) | | | | | | |  |
|  |  |  |  |  |  | CYR32 | CYR34 |  | CZ2016 | MY2016 | CZ2017 | MY2017 | CZ2018 | BLUP | |  | | CZ2016 | MY2016 | CZ2017 | MY2017 | CZ2018 | BLUP | |  | | CZ2016 | MY2016 | CZ2017 | MY2017 | CZ2018 | BLUP | |
| 133 | AS661845 | Yugongdiao | 2 | Shaanxi |  | 3 | 3 |  | 3 | 3 | 2 | 3 | 3 | 2.77 | |  | | 40 | 60 | 5 | 80 | 9 | 37.42 | |  | | 3.15 | 8.40 | 0.70 | 3.05 | 0.67 | 2.99 | |
| 134 | AS661848 | Canlaomai | 1 | Shaanxi |  | 4 | 4 |  | 0 | 0 | 0 | 0 | 1 | 0.44 | |  | | 0 | 0 | 0 | 0 | 7 | 5.04 | |  | | 0.70 | 0.00 | - | 0.00 | 0.53 | 0.58 | |
| 135 | AS661890 | Fengxiangmai | 2 | Gansu |  | 3 | 3 |  | 0 | 0 | 0 | 0 | 1 | 0.44 | |  | | - | 0 | 0 | 0 | 4 | 6.04 | |  | | - | 0.00 | 0.00 | 0.00 | 0.14 | 0.43 | |
| 136 | AS661891 | Baimai | 2 | Gansu |  | 3 | 3 |  | 3 | 3 | 2 | 1 | 3 | 2.42 | |  | | 60 | 60 | 9 | 13 | 12 | 29.81 | |  | | 2.63 | 3.15 | 0.77 | 0.63 | 0.60 | 1.59 | |
| 137 | AS662020 | Molengmai | 2 | Yunnan |  | 3 | 3 |  | 3 | 1 | 2 | 2 | 3 | 2.24 | |  | | 60 | 5 | 14 | 40 | 72 | 38.07 | |  | | 7.00 | 1.23 | 1.79 | 1.68 | 5.29 | 3.36 | |
| 138 | AS662021 | Duanmangmai | 1 | Yunnan |  | 3 | 3 |  | 0 | 0 | 0 | 0 | 0 | 0.24 | |  | | 0 | 0 | 0 | 0 | 0 | 3.59 | |  | | 0.53 | 0.00 | 0.00 | 0.00 | 0.00 | 0.34 | |
| 139 | AS662056 | Baiyumai | 2 | Gansu |  | 4 | 3 |  | 3 | 3 | 3 | 1 | 3 | 2.60 | |  | | 60 | 60 | 30 | 3 | 8 | 30.52 | |  | | 5.25 | 7.70 | 2.31 | 0.28 | 0.43 | 2.98 | |
| 140 | AS662057 | Baizaomai | 1 | Gansu |  | 4 | 4 |  | 1 | 0 | 0 | 0 | 1 | 0.61 | |  | | 5 | 0 | 1 | 0 | 8 | 6.18 | |  | | 0.18 | 0.00 | 0.28 | 0.00 | 0.56 | 0.45 | |
| 141 | AS662061 | Youmangyangmai | 2 | Gansu |  | 3 | 4 |  | 2 | 3 | 3 | 1 | 3 | 2.42 | |  | | 20 | 60 | 19 | 8 | 2 | 22.18 | |  | | 1.05 | 2.98 | 1.26 | 0.35 | 0.14 | 1.24 | |
| 142 | AS662062 | Hongchuanmai | 1 | Gansu |  | 4 | 4 |  | 1 | 0 | 0 | 0 | 1 | 0.61 | |  | | - | 0 | 0 | 0 | 1 | 5.31 | |  | | - | 0.00 | 0.35 | 0.00 | 0.46 | 0.57 | |
| 143 | WH037 | Chaoanxiaomai | 2 | Guangdong |  | 3 | 3 |  | 3 | 3 | 3 | 4 | 4 | 3.32 | |  | | 60 | 60 | 44 | 72 | 96 | 63.23 | |  | | 3.85 | 6.48 | 5.25 | 3.68 | 12.04 | 6.07 | |

^a^ underline indicates the materials can be recommended as crossing parents for resistance breeding

^b^ underline indicates the materials maintained stable high-level resistance to stripe rust in five environments
