# Supplementary material for: Genome-wide association mapping reveals potential novel loci controlling stripe rust resistance in a Chinese wheat landrace diversity panel from the southern autumn-sown spring wheat zone
Source: BMC Genomics. 2021 Jan 7;22:34. doi: 10.1186/s12864-020-07331-1 (PMC7791647; doi:10.1186/s12864-020-07331-1)
Supplement: Supplementary file 2 — Additional file 2. Pearson’s correlation coefficients for infection type (IT), final disease severity (FDS) and area under the disease progress curve (AUDPC) against stripe rust evaluated among five environments during 2016 to 2018. Different environments were all correlated, Significant at P < 0.01. [file 12864_2020_7331_MOESM2_ESM.pptx]

## Slide 1
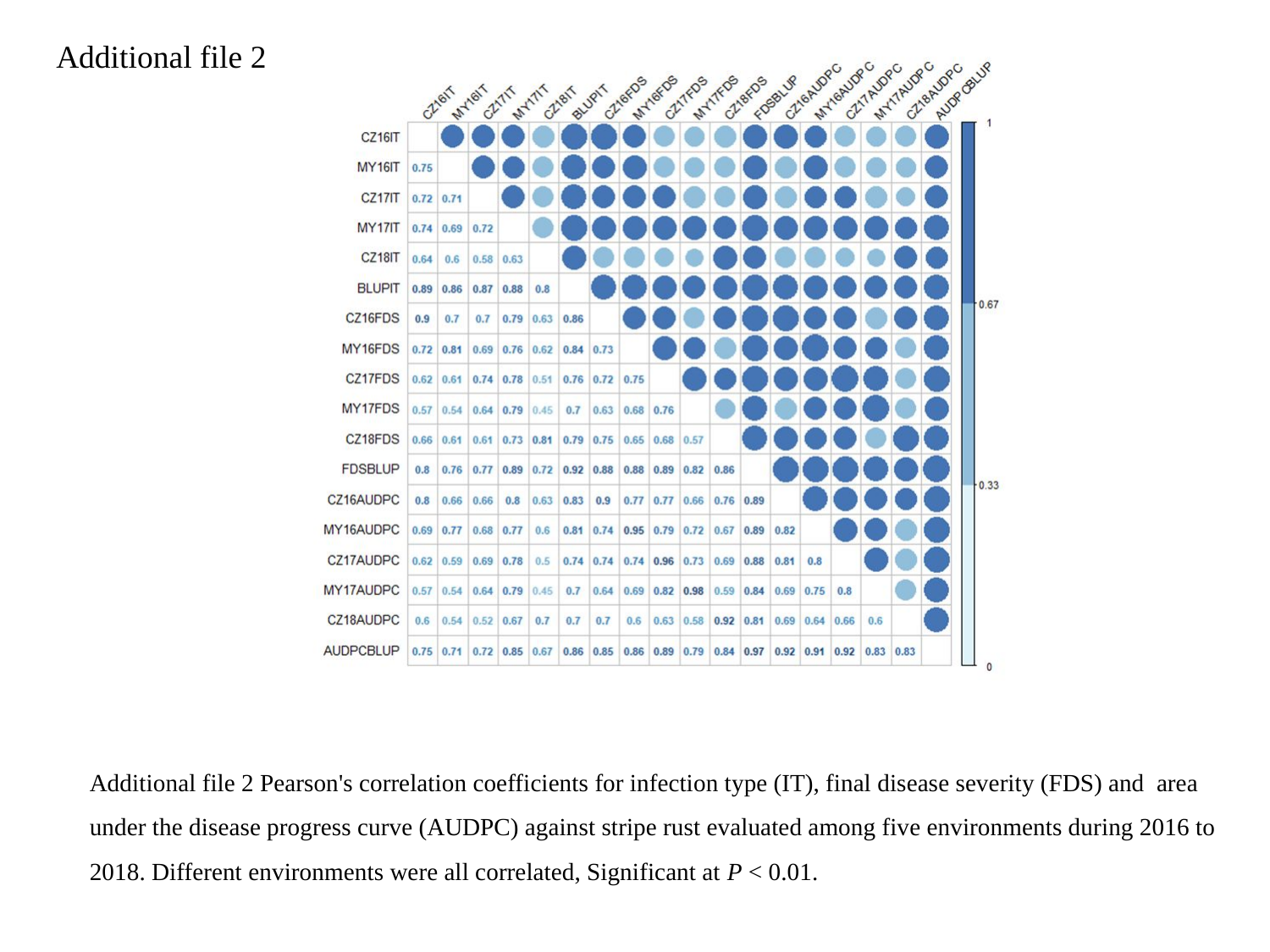

Additional file 2
Additional file 2 Pearson's correlation coefficients for infection type (IT), final disease severity (FDS) and area under the disease progress curve (AUDPC) against stripe rust evaluated among five environments during 2016 to 2018. Different environments were all correlated, Significant at P < 0.01.
