# Supplementary material for: Genome-wide association mapping reveals potential novel loci controlling stripe rust resistance in a Chinese wheat landrace diversity panel from the southern autumn-sown spring wheat zone
Source: BMC Genomics. 2021 Jan 7;22:34. doi: 10.1186/s12864-020-07331-1 (PMC7791647; doi:10.1186/s12864-020-07331-1)
Supplement: Supplementary file 3 — Additional file 3. Population structure of 143 wheat landrace accessions in Southern Autumn-Sown Spring Wheat Zone of China. (a) The population structure of 143 accessions with Bayesian clustering analysis. Two colors stand for 2 different compositions. The Subgroup 1 (Gp1) mainly showed as red color. The Subgroup 2 (Gp2) mainly showed as green color; (b) Estimated the distance of hierarchical clustering for the accessions using Fast Ward grouping algorithm and heat map showing the kinship and phylogenetic relations. [file 12864_2020_7331_MOESM3_ESM.pptx]

## Slide 1
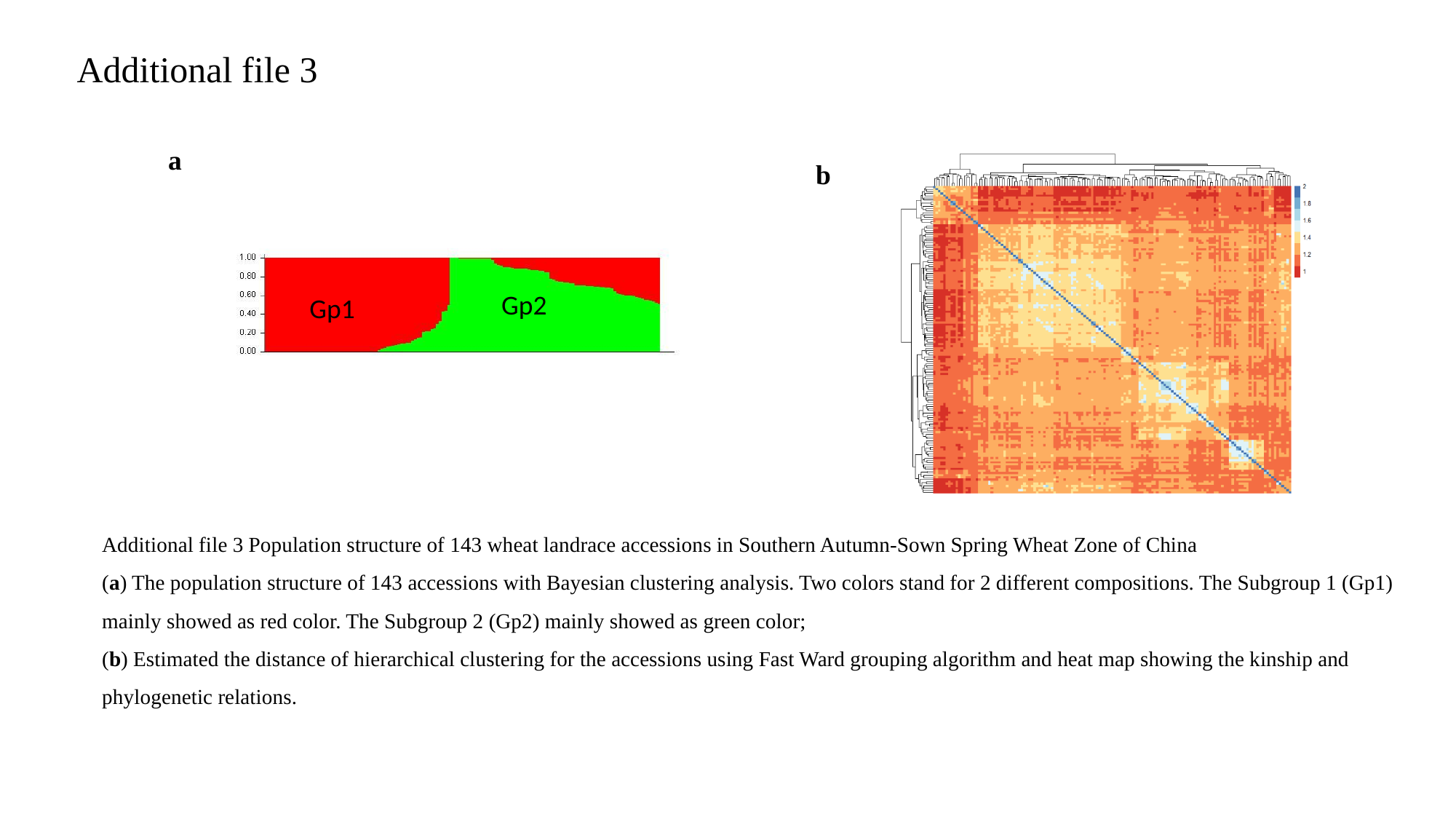

Additional file 3
a
b
Gp2
Gp1
Additional file 3 Population structure of 143 wheat landrace accessions in Southern Autumn-Sown Spring Wheat Zone of China
(a) The population structure of 143 accessions with Bayesian clustering analysis. Two colors stand for 2 different compositions. The Subgroup 1 (Gp1) mainly showed as red color. The Subgroup 2 (Gp2) mainly showed as green color;
(b) Estimated the distance of hierarchical clustering for the accessions using Fast Ward grouping algorithm and heat map showing the kinship and phylogenetic relations.
