# Supplementary material for: Genome-wide association mapping reveals potential novel loci controlling stripe rust resistance in a Chinese wheat landrace diversity panel from the southern autumn-sown spring wheat zone
Source: BMC Genomics. 2021 Jan 7;22:34. doi: 10.1186/s12864-020-07331-1 (PMC7791647; doi:10.1186/s12864-020-07331-1)
Supplement: Supplementary file 4 — Additional file 4. Genome-wide average linkage disequilibrium (LD) decay plot for 143 wheat landraces based on 5899 DArT markers. The scatter plots showing pairwise DArT markers LD r2 value as a function of inter-marker genetic distances (cM). [file 12864_2020_7331_MOESM4_ESM.pptx]

## Slide 1
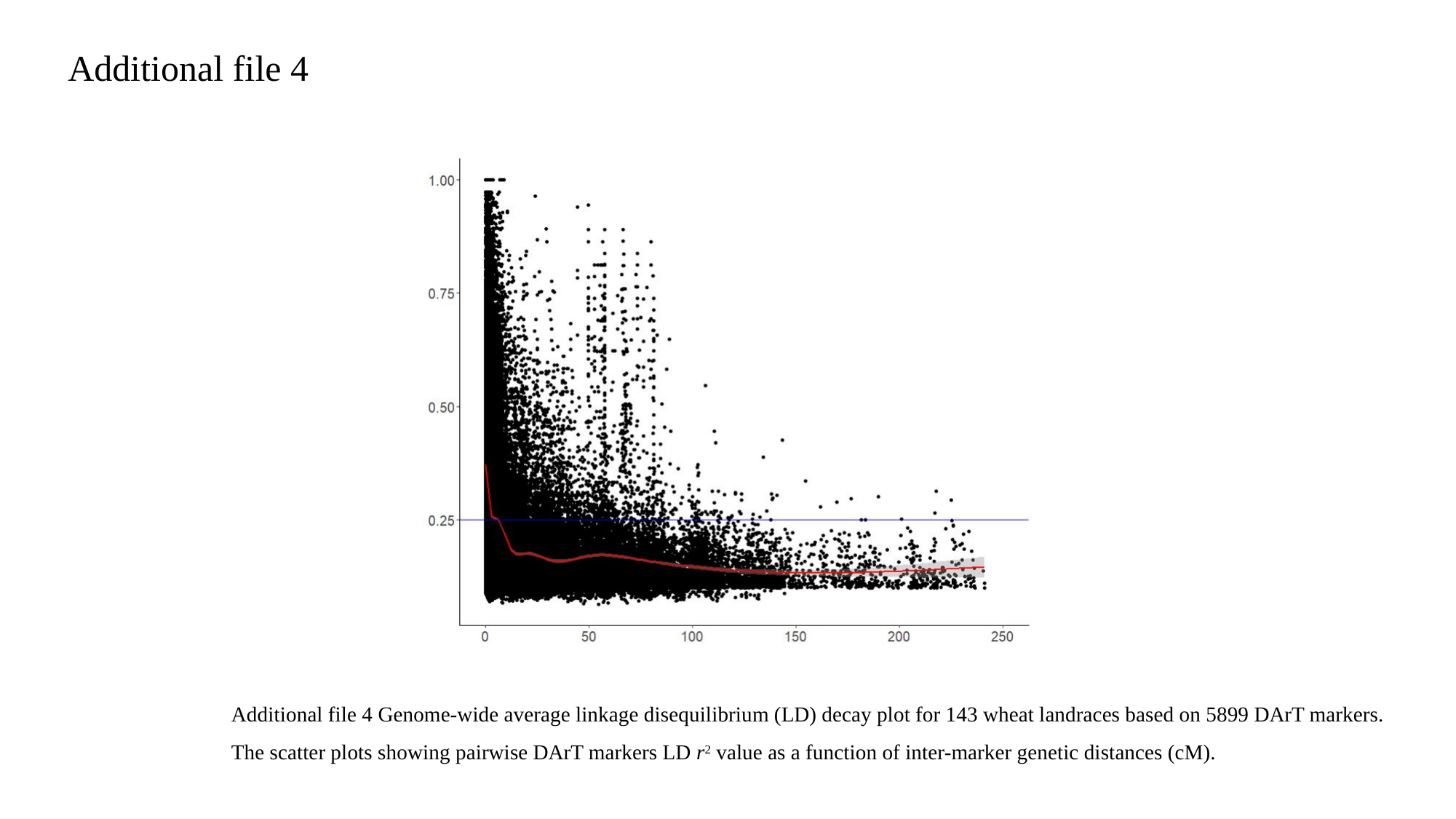

Additional file 4
Additional file 4 Genome-wide average linkage disequilibrium (LD) decay plot for 143 wheat landraces based on 5899 DArT markers. The scatter plots showing pairwise DArT markers LD r2 value as a function of inter-marker genetic distances (cM).
