# Supplementary material for: Genome-wide association mapping reveals potential novel loci controlling stripe rust resistance in a Chinese wheat landrace diversity panel from the southern autumn-sown spring wheat zone
Source: BMC Genomics. 2021 Jan 7;22:34. doi: 10.1186/s12864-020-07331-1 (PMC7791647; doi:10.1186/s12864-020-07331-1)
Supplement: Supplementary file 6 — Additional file 6. The avirulence(A) /virulence(V) formula of the Pst races used in this study. [file 12864_2020_7331_MOESM6_ESM.docx]

| **Additional file 6 The avirulence(A) /virulence(V) formula of the *Pst* races used in this study** | |  |
| --- | --- | --- |
| Race name | Virulence or avirulence formula on *Yr* genes | References |
| CYR32 | V: *Yr1,Yr2,Yr3,Yr4,Yr6,Yr7,Yr8,Yr9,Yr11,Yr12,Yr13,Yr14,Yr16,Yr17,Yr18,Yr25,Yr27,Yr28,Yr29,* | [9,37,75-77,79,80] |
|  | *Yr30,Yr31,Yr32,Yr43,Yr44,YrExp2,YrSp,Yr76,YrV23,YrA* |  |
|  | A: *Yr5,Yr10,Yr15,Yr19,Yr24,Yr26,Yr32,YrTr1,YrTye* |  |
| CYR33 | V: *Yr1,Yr2,Yr3,Yr4,Yr6,Yr7,Yr8,Yr9,Yr11,Yr12,Yr13,Yr14,Yr16,Yr17,Yr18,Yr25,Yr27,Yr28,Yr29,* | [75-77,79] |
|  | *Yr30,Yr31,Yr32,Yr43,Yr44,YrExp2,YrA,YrSk,YrSp* |  |
|  | A: *Yr5,Yr10,Yr15,Yr19,Yr24,Yr26,Yr32,YrTr1* |  |
| CYR34 | V: *Yr1,Yr2,Yr3,Yr4,Yr6,Yr7,Yr8,Yr9,Yr10,Yr11,Yr12,Yr13,Yr14,Yr16,Yr17,Yr18,Yr19,Yr24,Yr25,* | [24,37,75,77,79] |
|  | *Yr26,Yr27,Yr28,Yr29,Yr30,Yr31,Yr32,Yr43,Yr44,YrExp2,YrA,YrSk,YrSp,YrTye* |  |
|  | A: *Yr5,Yr15,Yr32,YrTr1* |  |
| G22–14 | V: *Yr1,Yr2,Yr3,Yr4,Yr6,Yr7,Yr8,Yr9,Yr10,Yr11,Yr12,Yr13,Yr14,Yr16,Yr19,Yr20,Yr24,Yr26* | [9,78] |
|  | A: *Yr3b,Yr4b,Yr5,Yr15,Yr17,Yr18* |  |
| Su11–4 | V: *Yr1,Yr2,Yr3,Yr4,Yr6,Yr7,Yr8,Yr9,Yr13,Yr14,Yr16,Yr18,Yr28,Yr29,Yr31,Yr43,Yr44,YrExp2* | [76,77,79] |
|  | A: *Yr5,Yr10,Yr11,Yr12,Yr15,Yr17,Yr19,Yr24,Yr26,Yr27,Yr32,YrSp,YrTr1* |  |
| Su11–5 | V: *Yr1,Yr2,Yr3,Yr4,Yr6,Yr7,Yr8,Yr9,Yr11,Yr14,Yr16,Yr18,Yr28,Yr29,Yr31,Yr43,Yr44,YrExp2,YrSp* | [76,79] |
|  | A: *Yr5,Yr10,Yr12,Yr13,Yr15,Yr17,Yr19,Yr24,Yr26,Yr27,Yr32,YrTr1* |  |
| Su11–7 | V: *Yr1,Yr2,Yr3,Yr4,Yr6,Yr7,Yr8,Yr9,Yr11,Yr12,Yr14,Yr16,Yr17,Yr18,Yr28,Yr29,Yr31,Yr43,Yr44,YrExp2,YrSp* | [76,79] |
|  | A: *Yr5,Yr10,Yr13,Yr15,Yr17,Yr19,Yr24,Yr26,Yr27,Yr32,YrTr1* |  |
